# scMaui: a widely applicable deep learning framework for single-cell multiomics integration in the presence of batch effects and missing data

**DOI:** 10.1186/s12859-024-05880-w

**Published:** 2024-08-06

**Authors:** Yunhee Jeong, Jonathan Ronen, Wolfgang Kopp, Pavlo Lutsik, Altuna Akalin

**Affiliations:** 1 Division of Cancer Epigenomics, German Cancer Research Center (DKFZ), Im Neuenheimer Feld 280, Heidelberg, Germany; 2 Faculty of Mathematics and Informatics, Heidelberg University, Im Neuenheimer Feld 205, Heidelberg, Germany; 3 Bioinformatics and Omics Data Science Platform, Max Delbrück Center for Molecular Medicine in the Helmholtz Association, Berlin Institute for Medical Systems Biology, Berlin, Germany; 4 Department of Oncology, Catholic University (KU) Leuven, Leuven, Belgium; 5 Roche Diagnostics GmbH, Penzberg, Germany; 6 Inceptive Nucleics, Inc., Palo Alto, CA USA

**Keywords:** Deep learning, Multi-omics, Single cell, Autoencoders

## Abstract

The recent advances in high-throughput single-cell sequencing have created an urgent demand for computational models which can address the high complexity of single-cell multiomics data. Meticulous single-cell multiomics integration models are required to avoid biases towards a specific modality and overcome sparsity. Batch effects obfuscating biological signals must also be taken into account. Here, we introduce a new single-cell multiomics integration model, Single-cell Multiomics Autoencoder Integration (scMaui) based on variational product-of-experts autoencoders and adversarial learning. scMaui calculates a joint representation of multiple marginal distributions based on a product-of-experts approach which is especially effective for missing values in the modalities. Furthermore, it overcomes limitations seen in previous VAE-based integration methods with regard to batch effect correction and restricted applicable assays. It handles multiple batch effects independently accepting both discrete and continuous values, as well as provides varied reconstruction loss functions to cover all possible assays and preprocessing pipelines. We demonstrate that scMaui achieves superior performance in many tasks compared to other methods. Further downstream analyses also demonstrate its potential in identifying relations between assays and discovering hidden subpopulations.

**Supplementary Information:**

The online version contains supplementary material available at 10.1186/s12859-024-05880-w.

## Introduction

Recent progress in high-throughput sequencing technology made it possible to profile different omics modalities from the same cells [1], so-called single-cell multiomics. For instance, CITE-Seq enables joint profiling of transcriptomes and proteomes [2], while single-cell nucleosome, methylation, and transcription sequencing (scNMT-seq) simultaneously yield chromatin accessibility, methylome, and transcriptome from the same cell [3].

Single-cell multiomics analysis broadens our perspective on cellular diversity which is important for numerous biological processes including embryonic development and ageing. The cellular diversity, which refers to different cell populations and subpopulations, has direct clinical implications for both diagnosis and treatment of diseases. Different distributions of cell subpopulations within the tumour microenvironment, for instance, are key components for the prediction of immunotherapy response and gene discovery [4–6]. The increasing quantity of spatial omics data emphasises the importance of single-cell multiomics analysis especially in cancer research [7].

In order to handle this complexity, various computational tools have been developed to integrate single-cell multiomics data and analyse cellular heterogeneity based on the integration of omics modalities [8–10]. These analyses can address many unanswered questions in biology and medicine, such as novel cell-type detection or relationships between different cells [11]. Seurat, one of the broadly used multiomics analysis R toolkits, contributed to revealing renal cell carcinoma regulatory programmes, as well as new cellular states in type I diabetes via single-cell multiomics analyses [12, 13]. Integration of single-cell gene expression, DNA methylation, and chromatin accessibility using MOFA unveiled the epigenetic landscapes during gametogenesis [14].

A variational autoencoder (VAE) is a deep generative model for mapping large input data to a low-dimensional latent space [15]. With the growing availability of single-cell multiomics data, VAEs have become a promising approach to summarise extremely complex and sparse datasets using interpretable latent factors [9, 16, 17]. VAE-based single-cell multiomics integration models differ according to the integrating approaches of multi-modalities. There are two common strategies of integration: product-of-experts (PoE) and mixture-of-experts (MoE). The PoE approach multiplies the density function of all modalities to build a joint distribution, whereas the MoE approach explains the joint posteriors as a summation of weighted density functions. Both of those have been applied to multiomics data integration [18–21]. Compared to the MoE approach, the PoE approach is able to generate a better joint posterior distribution from incomplete multi-modal data and has shown higher performance in predicting the mortality and drug sensitivity from the Cancer Genome Atlas (TCGA) dataset [20]. According to the benchmarking by Brombacher et al., the PoE-based method Cobolt performed better than MoE-based scMM in both biological preservation and trajectory conservation especially when the dataset contains a high number of cells [22].

Although VAEs are frequently used in single-cell multiomics integration, methods published thus far have significant limitations when applied in real experimental settings. Other VAE-based single-cell multiomics integration methods are often applicable only for a specific experimental setup (Supplementary Table 3). For instance, totalVI is designed particularly for CITE-Seq and employs a negative binomial distribution for read counts [9], while scMVAE and cobolt use a zero-inflated negative binomial distribution, which is only suitable for the combination of RNA-seq and ATAC-seq data [17, 19].

Multiomics datasets often suffer from strong batch effects that impede appropriate downstream analyses. Unfortunately, batch effect removal is often overlooked in VAE-based single-cell multiomics integration models. For instance, Cobolt and scMM do not provide clear guidance as to how technical variations can be removed through the neural networks, but rather assume that the input data is already processed by another batch effect removal method or comes from a single batch [18, 19]. However, batch correction methods are not available for every assay type. In the case that a model deals with the batch effect problem, the most common approach is to assign a single vector for addressing the full batch provenance of all the experimental data [9, 23]. These model designs may not cover realistic experimental setups that involve more than one batch effect factor and restrict the usability of the models.

**Fig. 1:**
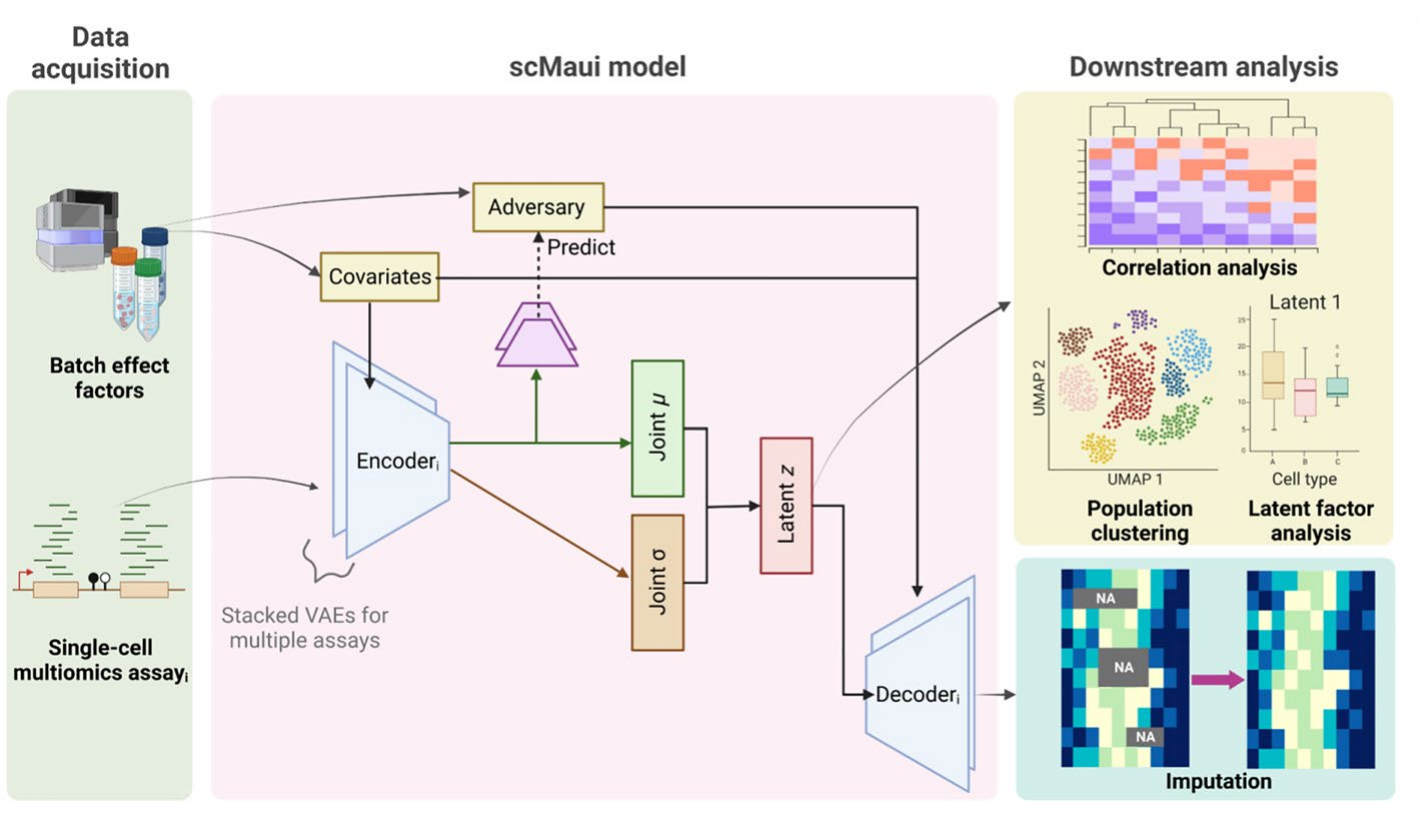
Illustration of scMaui model overview and the training process. Each single-cell multiomics assay is given to an encoder and batch effect factors are independently handled by covariates and adversary networks. Latent factors created by scMaui can be used for downstream analyses to find cellular heterogeneity (e.g. sub/population clustering) and reconstructed assays by the decoders can be used for imputation

Here, we present a new single-cell multiomics integration method based on variational PoE autoencoders, Single-cell Multiomics Autoencoder Integration (scMaui) to address the aforementioned limitations in VAE-based single-cell multiomics integration models (Fig. 1). scMaui can model all possible kinds of modalities with a flexible reconstruction loss function that supports varied probabilistic distributions including not only negative binomial but also Poisson, negative multinomial distributions, and many others. We also extended the batch-adversarial learning approach, which was introduced in our previous work [24], into the scope of multiomics data. Combining it with covariates reinforced the batch effect correction in scMaui algorithm. This new approach of batch effect handling allows users to flexibly assign batch labels and enables analyses of more complicated and realistic experimental designs.

We thoroughly assessed scMaui compared with other single-cell multiomics integration tools and conducted further biological analyses using various single-cell multiomics datasets including different assays. We show that scMaui outperforms other methods in many benchmarks, and is capable of cellular heterogeneity analysis across different biological samples. We also demonstrate the broad utility of scMaui through the analyses of diverse datasets and modalities.

## Results

### scMaui outperforms other methods in our systematic evaluation

We compared scMaui to other previously published methods in terms of cell population and subpopulation identification. Each of the integration methods produces a low-dimensional latent space representation of the data. We used a human bone marrow single-cell RNA-seq and antibody-derived tags (ADTs) dataset (GSE194122 [25]) as our main benchmarking dataset. Moreover, additional benchmarking was performed with SHARE-seq data including single-cell RNA-seq and ATAC-seq profiles from mouse skin cells [26]. Via this benchmarking, we show scMaui is not bound to a specific sequencing technology.

#### Cell population and subpopulation prediction

**Fig. 2:**
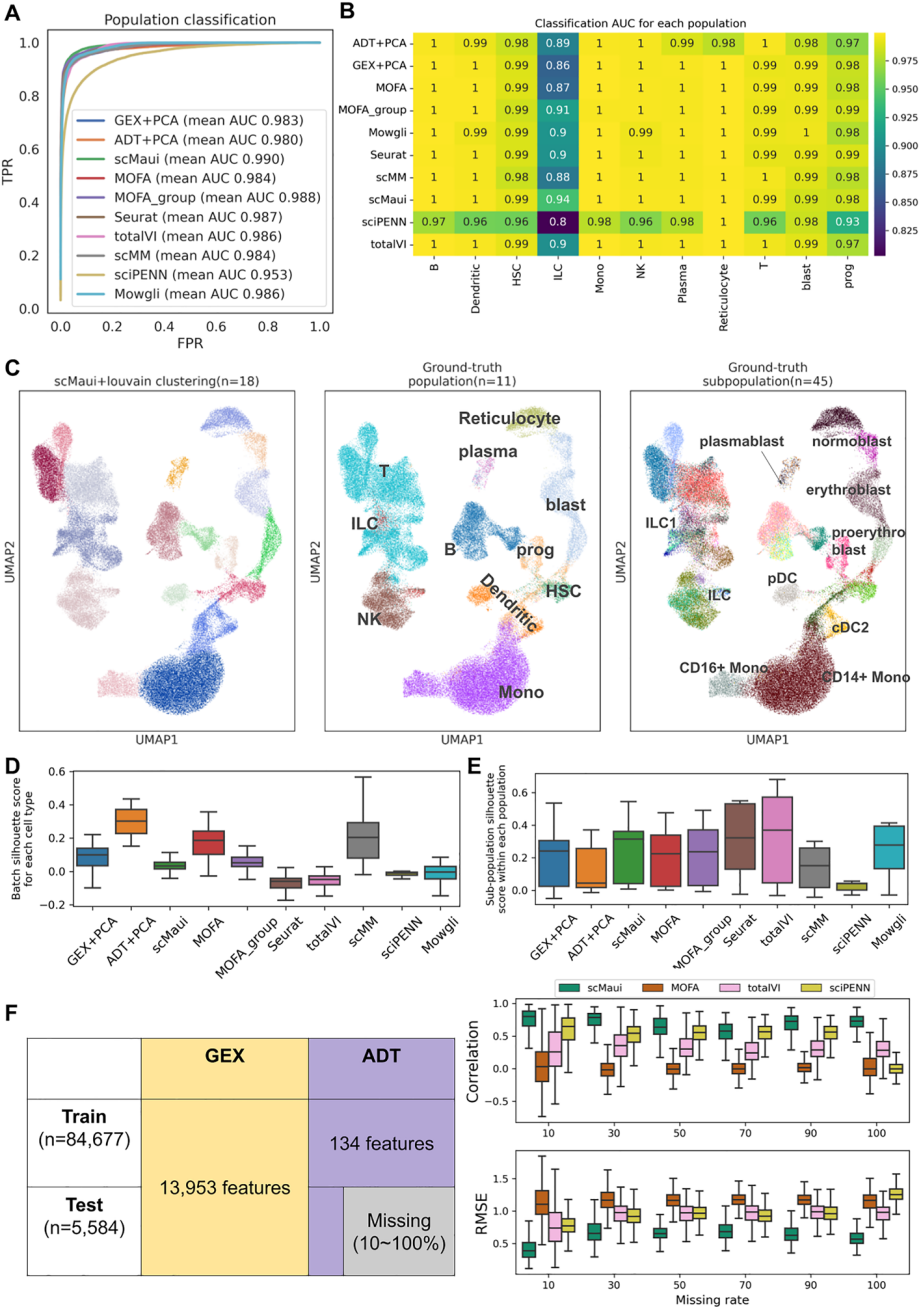
Benchmarking results of single-cell multiomics integration methods. **A** Cell population classification AUC-ROC curves and mean AUC. **B** Classification AUC value for each population and each method. **C** UMAP representation of scMaui latent factor coloured by clustering result, ground-truth population, and subpopulation labels. **D** Batch effect silhouette score in each subpopulation. **E** Subpopulation silhouette score in each population. **F** Protein expression (antibody-derived tags, ADT) modality imputation task dataset overview (left) and correlation results between predicted and ground-truth values. All boxplots present the median value as a middle bar in the box and both extremes are referred to as the first and the third quantiles

For the first benchmark, we sought to evaluate to what extent the cell-type heterogeneity is preserved in the low dimensional representation (cell population and subpopulation prediction). scMaui created 50 latent factors in this task. To that end, we trained classifiers to predict the cell population from the latent space representation, and calculated the area under the receiver operating characteristic curve (AUC-ROC) to measure classification performance. We used a support vector machine with tenfold cross validation and calculated average performance over all cell populations. Regarding average area under the curve (AUC), scMaui outperformed all alternative methods (Fig. 2A). The performance difference was most striking for the innate lymphocyte cell (ILC) population as shown in Fig. 2B. ILC population was particularly difficult to distinguish from T cells, because ILC and T cells share many molecular signals and regulate several similar immune functions [27]. However, ILCs do not have an adaptive T cell receptor and belong to the innate immune system [28], making them a distinct population which scMaui identified with better AUC than the other methods. We proceeded with the same analysis, but on the cell subpopulation labels (Supplementary Fig. 1). MOFA, both with and without the group option provided within the method itself, outperformed other methods showing high performance in the classical dendritic cell 1 (cDC1) subpopulation, but scMaui still achieved a better mean AUC score than Seurat and totalVI.

#### Clustering analysis

In practice, ground-truth cell-type labels are often not available for single-cell multiomics analysis. Therefore we also evaluated all methods in terms of clustering, rather than supervised classification. Louvain clustering was applied to the latent factors extracted by each method. After running Louvain clustering, we measured the clustering performance with adjusted mutual information (AMI), adjusted random index (ARI), and clustering purity (Table 1). To find the best clustering result for each method, 20 different resolution values (increased by 0.1 from 0.1 to 2.0) were tried (Supplementary Fig. 13), and the value recording the best AMI score was selected. scMaui achieved the highest score in all these clustering performance measurements. Figure 2C shows the UMAP plot of scMaui latent factors with Louvain clustering results, ground-truth population, and subpopulation respectively. In total, 18 clusters were detected via the Louvain clustering algorithm, which is between the number of populations and subpopulations. The detected clusters covered most ground-truth populations and some subpopulations. In particular, for subpopulations, individual clusters represent reticulocytes, three subpopulations of blasts, and two monocyte subpopulations most distinctly.

**Table 1:** Performance comparison with respect to clustering and dimensionality reduction

	Best resolution	AMI	ARI	Clustering purity	# Detected cluster	Population silhouette score
GEX+PCA	0.4	0.709	0.603	0.584	16	0.255
ADT+PCA	2.0	0.623	0.268	0.656	53	0.139
scMaui	0.7	**0.795**	**0.737**	**0.722**	18	0.274
MOFA	1.3	0.683	0.345	0.714	40	0.183
MOFA_group	0.4	0.752	0.703	0.658	18	0.157
Seurat	0.3	0.759	0.713	0.617	16	**0.312**
totalVI	0.9	0.772	0.542	0.710	25	0.296
scMM	0.9	0.649	0.315	0.647	29	0.151
sciPENN	0.7	0.266	0.228	0.362	10	0.008
Mowgli	0.3	0.743	0.559	0.656	15	0.212

Louvain clustering algorithm was applied to the low-dimensional latent factors/features extracted by each method. Clustering results were assessed with AMI, ARI, and clustering purity score, while the population silhouette score indicates how well-separated populations are in the extracted latent factors/features. The best value of each score is highlighted in bold.

The silhouette score measures how well-separated given sets of labels are within the feature space. We calculated the silhouette score for batch labels and population labels in the latent space created by each method. For batch labels, the score was computed within each subpopulation (Table 1 and Fig. 2D). While a high silhouette score is expected for well-separated populations, ideally batch-corrected latent space should record a low silhouette score with respect to the batch labels. Regarding population and batch silhouette scores, Seurat performed best, but scMaui showed relatively high performance compared to MOFA and PCA applied for each assay. We also confirmed that using both covariates and adversaries improves the batch correction while preserving the cell population prediction via an ablation study (Supplementary Table 5 and Fig. 10). We furthermore calculated silhouette scores of subpopulations within each population in order to investigate how well-distributed subpopulations are in their population cluster (Fig. 2E). scMaui achieved the third highest median subpopulation silhouette score following totalVI and Seurat.

#### Imputation

We also assessed scMaui and other methods in a missing data imputation task (Fig. 2F). Here, new test data was introduced from the same GEO database, but data from the protein expression modality was partially masked with different rates. For comparison, we included only MOFA and totalVI, which had a clear tutorial of protein expression imputation. scMaui imputed masked protein expression values most accurately showing the highest correlation and the lowest root mean squared error (RMSE) with respect to the ground-truth protein expression value, regardless of the missing rate. We performed the same imputation experiments on the gene expression modality and scMaui again achieved the best score for both the RMSE and the correlation regardless of the missing rate (Supplementary Fig. 7). When both gene expression and protein expression values were missing, scMaui could still estimate accurate values of both expression modalities (Supplementary Fig. 8). In this experiment, we set a missing rate of 30% for both modalities.

#### Application to SHARE-seq data

Simultaneous profiling of RNA-seq and ATAC-seq from the same cells elucidates interactions between transcriptome and epigenome at a single-cell level. Many technologies have been developed for the multiomic profiling of RNA-seq and ATAC-seq from the same cells (e.g., SHARE-seq, SNARE-seq, and scCAT-seq) [29]. Here, we showcase the broad applicability of scMaui together with competitive performances by conducting additional comparisons to other methods using mouse skin SHARE-seq data (Methods). Please, note that we replaced totalVI in the previous benchmarking with MultiVI [30] due to the inapplicability to RNA-seq and ATAC-seq integration. sciPENN is not included for the same reason. We also used different versions of Seurat [31, 32] for this benchmarking. The details are explained in Methods.

In the cell-type classification result (Supplementary Fig. 11), scMaui achieved the highest mean AUC value. scMaui latent factors made an accurate classification of cell types which were not accurately classified by other methods, such as macrophages or Schwann cells. When it comes to the Louvain clustering results (Supplementary Table 6), scMaui achieved the second-best clustering purity. The highest AMI and ARI clustering scores of GEX+PCA can be explained by the cell-type annotations assigned to clusters created using the RNA-seq modality according to the authors [26]. Supplementary Fig. 12 presents that the Louvain clusters detected with scMaui latent factors represent the majority of cell types with the exception of bulge cells and transit-amplifying cells (TACs). Overall, scMaui shows its competitive performance in cell-type identification using the integrated profile of RNA-seq and ATAC-seq, proving its broad applicability across multiomics technologies.

### scMaui is capable of trajectory inference and subpopulation examination in human bone marrow samples

Subpopulation examination and trajectory inference (e.g. for normal or diseased cell differentiation and maturation) are among the most frequent use cases of single-cell multiomics analysis [33–35]. Cell populations and subpopulations can be annotated based on marker gene expression referring to already published single-cell atlases, but inconsistent definitions of cell states and subpopulations between different reference datasets make it challenging to interpret single-cell multiomics data [36]. Another major challenge comes from the cells in transition between states. Transcriptomic signal change is continuous rather than discrete and cell fates can be decided stochastically [37]. These alterations need to be thoroughly described for further analyses such as trajectory inference, also known as pseudo-time ordering, which tracks the dynamic cell differentiation among single cells. Therefore, single-cell multiomics integration tools should be able to deal with the change of expression levels accordingly and extract features which can explain the transition of cell states based on multiple assays. We validated the usability of scMaui in these regards using single-cell RNA-seq and ATAC-seq from healthy bone marrow samples (GSE194122 [25]). Here, we used 63,138 cells provided by 9 donors and processed in 4 different sites. 10 populations including 22 subpopulations were annotated in the dataset. scMaui was set up to generate 50 latent factors as in the previous task.

**Fig. 3:**
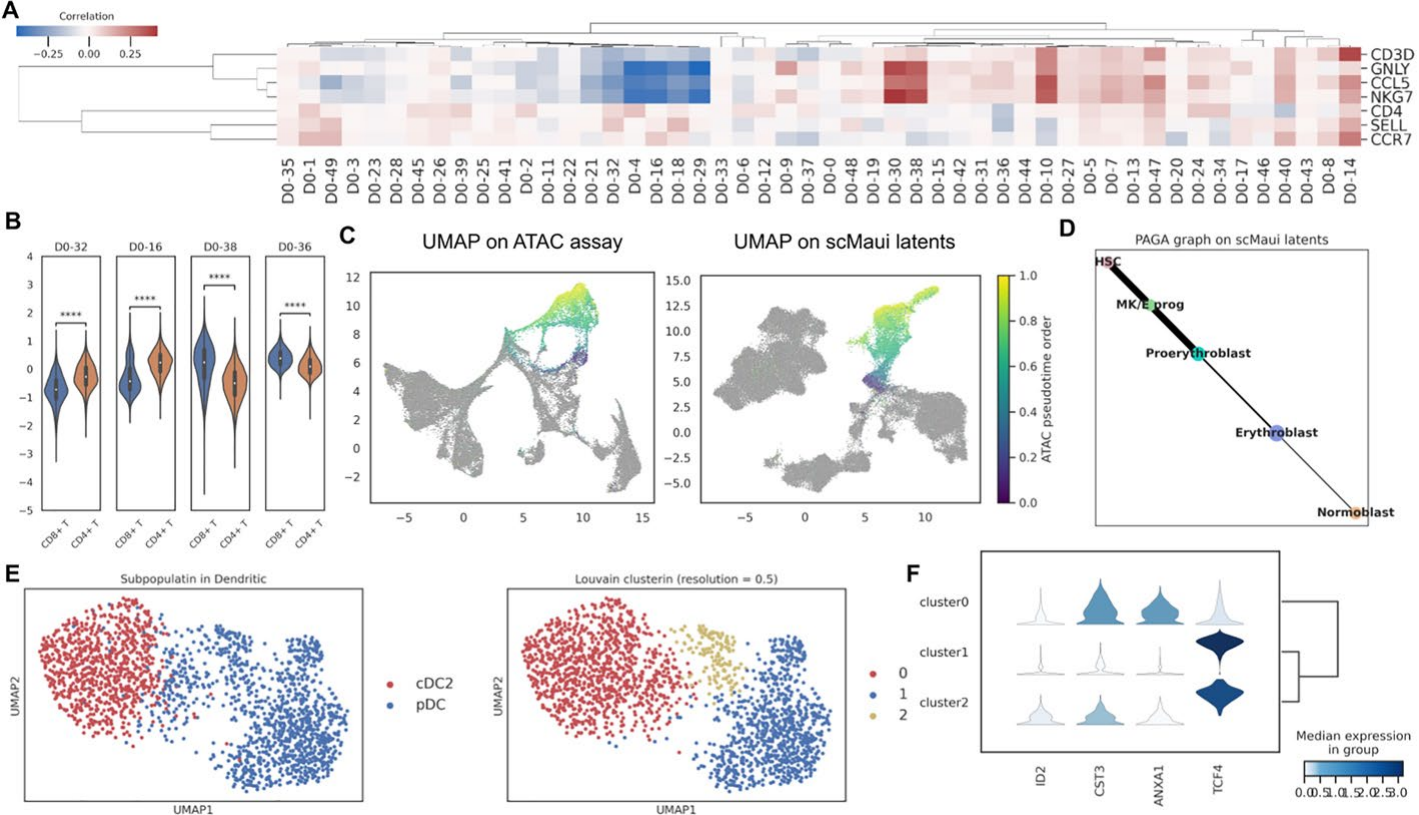
Subpopulation examination and cell-trajectory analyses using scMaui. **A** Correlation between 50 scMaui latent factors and T cell marker genes. **B** Distribution of latent values over CD4+ and CD8+T cells. Only latent factors highly correlated with their marker genes were chosen. **C** UMAP plot of PCA derived from ATAC-seq assay (left) and scMaui latent factors (right) coloured by ATAC-seq pseudo-time order. **D** PAGA graph applied to scMaui latent factor of HSC, MK/E progenitor, and blast subpopulations. **E** UMAP plot of scMaui latent factor coloured by dendritic subpopulations (left) and Louvain clusters (right). **F** Dendritic subpopulation marker gene expression analysis in the detected Louvain clusters

First, we calculated the correlation between latent value and some T cell marker gene expression (Fig. 3A). We selected *CD3D* as a marker for general T cells, *GNLY*, *CCL5*, *NKG7* as markers for CD8+T cells, and *CD4*, *SELL*, *CCR7* as makers for CD4+ cells based on previous studies [10, 25]. These 3 groups of markers resulted in the same 3 clusters based on the correlation, via hierarchical clustering, and the 3 CD8+T cell markers notably showed similar patterns of correlation values. Moreover, CD8+ cells had higher values for the latent factors 38 and 36 (Fig. 3B) which are also highly correlated with the CD8+T cell marker genes. On the other hand, CD4+T cells showed higher values than CD8+T cells at latent factors 32 and 16, which the CD4+T cell markers are highly correlated with. These results demonstrate that scMaui is capable of summarising cell-type-specific signals into its latent factors without prior knowledge about markers or cell types.

Figure 3C displays ATAC pseudo-time of hematopoietic stem cell (HSC), megakaryocytic-erythroid progenitors (MK/E prog), and blast cells on UMAP plots calculated on principal components (PCs) of ATAC-seq assay and on scMaui latent factors. In this dataset, the pseudo-time order of cells was generated in each modality using diffusion pseudo-time [38]. Compared to the UMAP computed directly from PCs, scMaui latent factors made a better distribution of single-cell data accordingly with ATAC pseudo-time order. A partition-based graph abstraction (PAGA) map generated from scMaui latent factors also reflects the order of cell development from HSC to normoblast [39] as a consistent result with the pseudo-time order (Fig. 3D). PAGA was developed to graph a topological structure in single-cell data points based on partition connectivity, and has been used for precise identification of biological trajectories [40]. We performed the same cell-trajectory analysis with MOFA and Seurat (Supplementary Fig. 2). While Seurat made a consistent result with scMaui, MOFA factors could not distinguish HSC, proerythroblasts, and normoblasts as clearly as scMaui did and created weak connections between HSC and two blast subpopulations.

In further analysis, scMaui detected 3 clusters (using Louvain clustering as described above) in plasmacytoid dendritic (pDC) cells, which are a subpopulation of dendritic cells (Fig. 3E). To explore the discrepancy between the detected 2 clusters in pDC, we investigated the expression level of some dendritic marker genes in all 3 clusters found in the dendritic population (Fig. 3F). Cluster 0 mostly belongs to cDC2 cells according to both UMAP plots and expression analysis. However, clusters 1 and 2 show a difference mainly in *ID2* and *CST3* genes, which are considered as cDC1 cell markers rather than pDC cell markers according to Lin et al. and Schlitzer et al. [41, 42]. *ID2* and *CST3* genes are generally hyper-expressed in cDC1 cells. Therefore, we presume that pDC cells in this dataset might include cDC1-like cells in cluster 2, although the dataset does not contain cDC1 in their cell-type labels. This provides further evidence of scMaui’s superior ability to differentiate between subtle cell subtypes without prior knowledge.

### scMaui can explain both mouse embryo development and cellular heterogeneity based on DNA methylation and gene expression

 DNA methylation dynamics during embryonic development leads to the emergence of cell-type-specific DNA methylation patterns [43]. In mammal genomes, cell-type-specific methylation profiles are shown especially at CpG sites. Due to its stability and inheritability, DNA methylation has been one of the most widely studied epigenetic modifications. Many computational methods have been published for DNA methylation analysis based on bisulfite sequencing (BS-seq) data [44]. However, data sparsity is particularly severe in single-cell methylation assays compared to other assays and can bias statistical models. In addition, methylation is also affected by gender, age, and environmental influences. Thus, single-cell multiomics integration should ideally be able to distinguish signatures derived from different factors, so that the desired variations under the relevant conditions for a study do not get mixed with technical and other biological sources of variation.

During the development of the mouse embryo, each embryonic stage and cell differentiation state differ in their transcriptomic and epigenetic landscapes. Hence, we analysed both transcriptomic and epigenetic assays of mouse embryo development using scMaui. The single-cell multiomics dataset was collected from GSE121708 [45]. After removing cells based on quality control (QC) results, we used 939 cells from 8 cell populations in the analyses. The cells were acquired from 28 different embryos in 4 different stages. When only the RNA-seq assay was analysed with PCA, strong batch effects were shown as epiblast and primitive streak cells tend to be clustered by samples rather than cell populations (Supplementary Fig. 3A and C). The UMAP of PCs coloured by embryo stage also poorly recapitulates the change of embryo stage (Supplementary Fig. 3B).

**Fig. 4:**
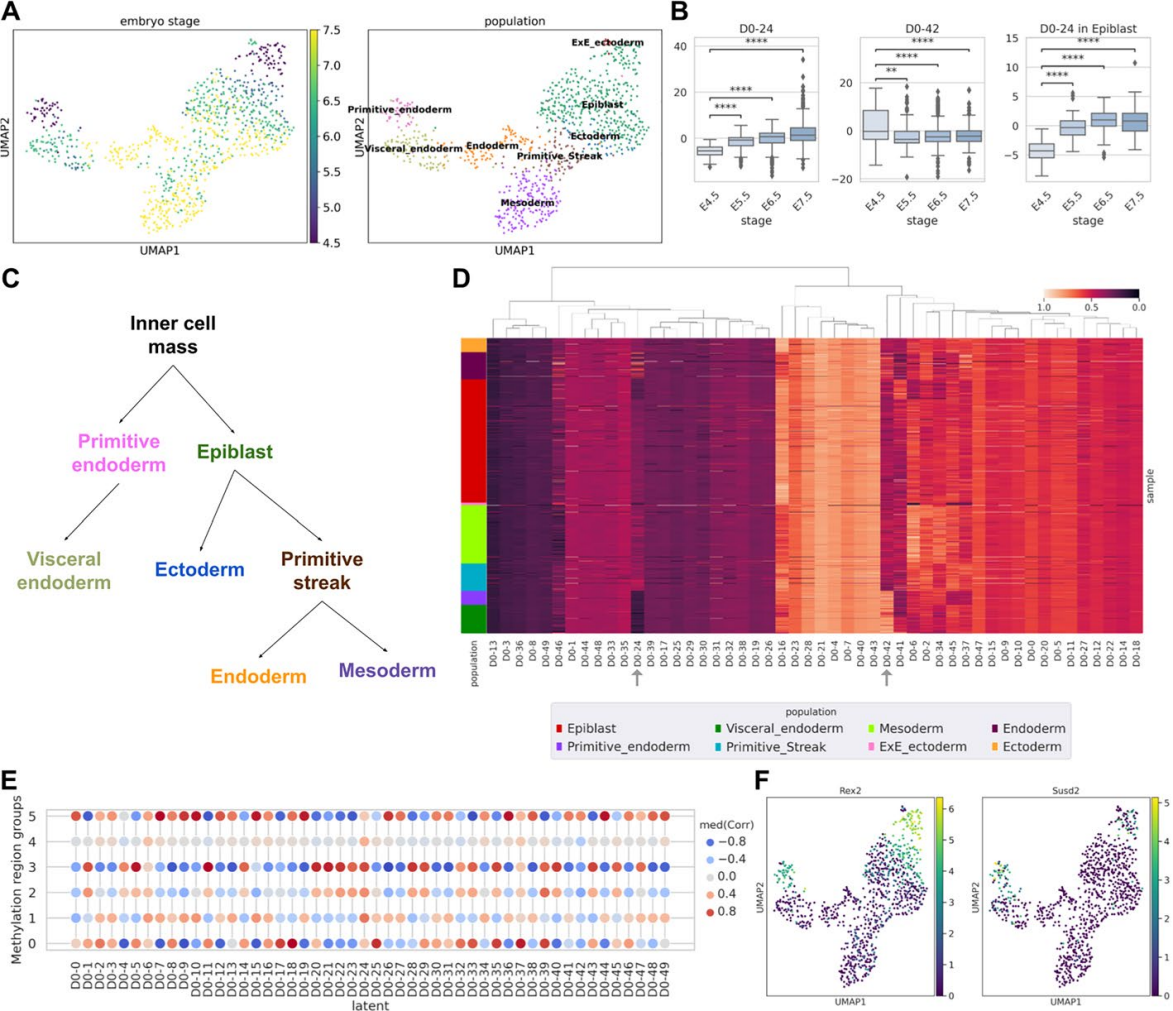
Mouse embryo single-cell gene expression and methylation multiomics data analysis results. **A** UMAP plot of scMaui latent factor extracted from the entire dataset. The plots are coloured by embryo stages and populations each. **B** Latent values in different stages of embryo cells. Latent factors 24 and 42 are presented at the left and the middle. The right boxplot shows the latent factor 24 values by embryo stage only in epiblast cells. **C** Diagram of embryonic cell developmental lineage. **D** Latent values normalised between 0 and 1 and ordered by population. **E** Median correlation between each latent factor and methylation level of each region group. The groups were decided based on clustering methylation levels. **F** Gene expression of *Rex2* and *Susd2* over all single-cell samples

On the other hand, scMaui could alleviate these issues and make a more meaningful organisation of cell populations (Fig. 4A right). scMaui created a latent space with 50 factors and could precisely distribute mouse embryo cells according to biological cell lineages [45–47] in Fig. 4C. We also found that scMaui latent factors could be used to detect embryo developmental stages (4.5, 5.5, 6.5 and 7.5) within respective cell populations (Fig. 4A left). For instance, the epiblast population includes all four different stages, and the cells were aligned along the UMAP2 axis accordingly. We did the same analyses with MOFA and Seurat (Supplementary Fig. 5) but neither could make a clear representation of embryo development stage changes as scMaui did. MOFA factors clearly distinguished between different stages but did not organise the clusters along the stage change. Seurat PCs could better arrange the cells according to the stage, but E4.5 cells are distributed together with other cells and are not very distinct.

Among the fifty latent factors extracted by scMaui, latent factor 24 explicitly reflects the mouse embryo development stages (Fig. 4B left). Although some epiblast cells present lower values than others in latent factor 24 (Fig. 4D), there is still a clear increase in the latent value from stage E4.5 to E5.5, and from E5.5 to E6.5 (Fig. 4B right). Latent factor 42 shows much higher values in the stage E4.5 cells than in the other stages of cells (Fig. 4B middle), and primitive and some visceral endoderm cells, which are in the E4.5 stage, also present very high values in latent factor 42 (Fig. 4D).

In order to evaluate whether scMaui can detect methylation signals according to cellular heterogeneity, we calculated the correlation between all latent values and methylation beta values in the promoter/enhancer regions selected as explained in Methods. We clustered the methylation regions into six groups using agglomerative clustering (Supplementary Fig. 6) and analysed genes assigned to the regions in groups 2 and 3. These groups were chosen due to the strong positive correlation with latent factor 24 and negative correlation with latent factor 42 (Fig. 4E). Figure 4F depicts the expression of the *Rex2* gene (assigned to chr4:147019856-147023856 region in group 2) and *Susd2* (assigned to chr10:75642008-75646008 region in group 3). Both are highly expressed in E4.5 cells (primitive endoderm and early epiblast populations). To sum up, cells with high expression of *Rex2* and *Susd2* have high values in latent factor 42 and methylation levels in promoter/enhancer regions present a strong negative correlation with latent factor 42, which means these regions are hypomethylated, reflecting the well-established inverse dependency between gene expression and methylation state of corresponding promoter and enhancer regions. Therefore, scMaui is capable of detecting a pair of relevant genes and methylation signals in terms of both mouse embryo development stages and cell-type heterogeneity.

## Discussion

 The number of studies using single-cell multiomics is rapidly growing due to the benefits of matched omics profiles from the same cells. However, the high complexity of single-cell multiomics data requires sophisticated computational tools to integrate assays into more comprehensive data representations, where cellular heterogeneity is clearly revealed and different sources of variation are disentangled. Different kinds of machine learning algorithms have been applied to this problem delivering better comprehension of molecular interactions between cells. Nonetheless, many of the methods are limited to specific assays or not designed to handle multiple batch effect factors.

In this study, we presented scMaui, a variational PoE autoencoders-based single-cell multiomics integration model available for unrestricted types of assay and batch effect factors. The PoE approach factorises the joint distribution into the product of marginal distributions from respective assays and has a strength in multi-modal modelling with incomplete data. In order to reflect the distribution of the different assays, scMaui supports multiple types of reconstruction errors. With regards to batch effect correction, scMaui handles multiple factors independently via separate feed-forward networks and adversarial learning. Considering that different batch effect factors (e.g. patients, pipelines) introduce independent and irrelevant variations to data, alleviating different sources of variation separately in single-cell multiomics data, as scMaui does, is statistically more coherent.

**Fig. 5:**
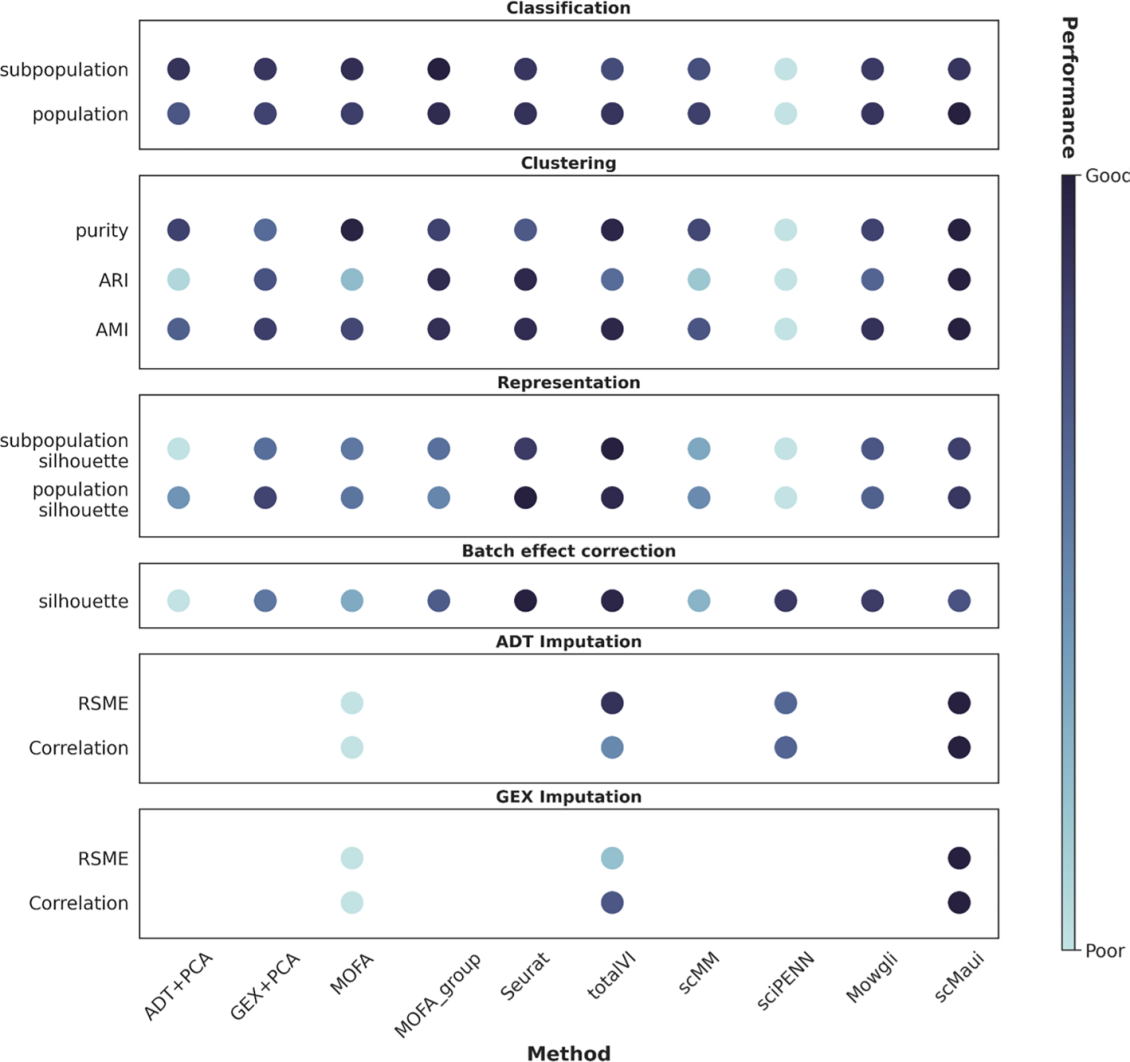
Summary of evaluation results conducted in our study. The darker the colour is, the better the method performed in each task. Performance scores are normalised by dividing with the standard deviation in each task

We assessed scMaui compared to several previously published methods. Single-cell RNA-seq and protein expression data collected from human bone marrow samples were used for the evaluation. A summary of the evaluation is presented in Fig. 5. scMaui outperformed other methods in population classification, population clustering, and protein expression imputation tasks. Through the classification AUC values for each population, we showed that scMaui performs particularly well for cell populations sharing similar expression signals. For the cell population clustering analysis, scMaui recorded the best AMI, ARI, and clustering purity scores. Finally, due to the advantage of the PoE approach, scMaui greatly outperformed all other methods in the imputation task, predicting much more accurate protein expression data regardless of how much of the data the model was shown.

We conducted further downstream analyses of two more single-cell multiomics datasets with scMaui. Latent factors extracted from bone marrow single-cell RNA-seq and ATAC-seq dataset captured constant patterns of expression within CD4+ and CD8+T cell marker gene groups respectively. Also, we were able to identify latent factors highly correlated with marker genes that presented higher values only in the corresponding cell type. These results indicate that scMaui can create its latent space corresponding to cell-type heterogeneity. scMaui latent space also showed a better organisation of HSC, MK/E progenitor, and blast cells according to ATAC-seq pseudotime compared to MOFA factors and made a clear cell trajectory inference result. Lastly, based on the clustering result on scMaui latent factors, we found a new potential subpopulation in dendritic cells showing expression levels more similar to cDC1 cell type which is not annotated in the metadata.

Analysis of the DNA methylome provides an insightful perspective of epigenomic phenomena which induce different levels of gene expression. scMaui was able to create latent factors summarising relevant features in gene expression and methylation assays together, with a single-cell mouse embryo multiomics dataset. Compared to other previous methods, the UMAP representation of scMaui latent factors arranged cells appropriately for both embryo development stages and cell populations. Also, we discovered that some scMaui latent factors can reflect the nature of molecular interactions between DNA methylation and gene expression, such as gene silencing in response to hyper-methylation at CpG sites within promoter or enhancer regions.

In conclusion, scMaui not only achieved better performance in many different analyses compared to other single-cell multiomics integration methods, but also revealed cellular heterogeneity and subpopulations that had been hidden in individual omics layers. From the technical standpoint, high flexibility supporting different types of reconstruction loss functions, separated batch effect handling and missing feature indicator are distinct benefits of scMaui. Thanks to this flexibility, scMaui covers more varied datasets produced via different workflows than other methods designed for specific assays. Last but not least, scMaui provides a user-friendly and modern API relying on standard data structures. Consequently, we believe that the usability and accessibility of scMaui will extend its usage to a broader range of researchers.

Although scMaui has shown outstanding performance and the ability to analyse cellular heterogeneity, some scenarios still need to be optimised. Because of the high cost of multi-assay measurements and numerous single-cell data already processed and stored in biobanks, Campbell et al. and Amodio et al. attempted to integrate multiple layers of omics data collected from similar yet different cells [48, 49]. This task mainly tries to create an integrated embedding space covering multiple assays and match clinically related cells between different assays. Since scMaui can process multiple modalities simultaneously and integrate those into a common embedding space, it might be possible to extend scMaui to multiomics data collected from unmatched cells or samples.

## Methods

### scMaui model description

#### Variational product-of-experts autoencoders

scMaui assigns a pair of encoder and decoder to each modality as illustrated in Fig. 1, so that assay-specific features can be extracted. In order to create a joint embedding space for all modalities, scMaui deploys additional layers calculating the joint mean and standard deviation of encoded features from all assays based on the PoE approach. We assume that the embedding representation of each assay follows a Gaussian distribution, thus the joint representation calculated as the production of marginal distributions will also be a Gaussian distribution. Building on the approximation of true joint posterior introduced by Wu et al. [50], the joint standard deviation (${\sigma }_{joint}$) and joint mean (${\mu }_{joint}$) for $N$ assays are calculated as below:

$${\sigma }_{joint} = ({{{\sigma }_{0}}^{-1}+}{\sum }_{i=1}^{N}{{\sigma }_{i}}^{-1}{)}^{-1}$$

$${\mu }_{joint}= ({\mu }_{0}{{{\sigma }_{0}}^{-1}+}{\sum }_{i=1}^{N}{{{\mu }_{i}{\sigma }_{i}}}^{-1})({{{\sigma }_{0}}^{-1}+}{\sum }_{i=1}^{N}{{\sigma }_{i}}^{-1}{)}^{-1}$$

${\mu }_{0}$ and ${\sigma }_{0}$ are two parameters of the prior Gaussian distribution explaining the joint embedding space, and we set it to a standard Gaussian distribution, $N(0, {\rm I})$.

#### Flexible batch effects removal using adversarial learning and covariates

scMaui excludes sources of variations introduced by batch effects using two different techniques. Firstly, vectors storing batch effect factors are fed into the encoders and decoders individually as covariates. Secondly, scMaui uses adversarial learning via additional feed-forward networks and gradient reversal layers [51]. The decoders reconstruct the input assays only from the joint latent factors and batch effect factor information conveyed by adversaries and covariates respectively.

Flexibility in batch effect correction is one of the strengths of scMaui. Batch effect factors can be both categorical and continuous values. Although varied statistical methods for batch effect correction have been proposed [52, 53], these commonly allow only categorical batch effect factor values or even require further preprocessing such as separation of input dataset by batches. scMaui supports automatic and simultaneous correction of multiple batch effects [24].

Deep neural networks are optimised in a direction towards decreasing the given loss function value over multiple steps. scMaui has a loss function which consists of three terms:

1 $${L}_{total} = {{L}_{kl}({\mu }_{joint}, {\sigma }_{joint} ) +\Sigma }_{i =1...I }({L}_{recon}({Y}^{i}, \widehat{{Y}^{i}})) - {{\Sigma }_{j=1...J}{ {L}_{adv}({b}^{j}), }}$$

where each assay ${Y}^{i}\in {\mathbb{R}}^{batch\_size \times N}$ has $N$ features. In scMaui, varied types of reconstruction loss (${L}_{recon}$) functions are supported depending on the assay type and the distribution of expression levels. For instance, a negative binomial loss function is reasonable to read count assays whereas methylation data can be better reconstructed by a binary loss function owing to the bimodal distribution of methylation beta values. A full list of supported reconstruction losses is available in Supplementary Methods.

For each VAE assigned to an assay $i$, KL-divergence loss (${L}_{kl}$) is calculated as generally done in VAE training [54]:

$${L}_{kl}\left({\mu }_{joint}, {\sigma }_{joint}\right)= -\frac{{w}_{kl}}{2}\left({\Sigma }_{l=1...K}\left(1+log\left({{{\sigma }^{l}}_{joint}}^{2}\right)-{{{\mu }^{l}}_{joint}}^{2} -{{{\sigma }^{l}}_{joint}}^{2}\right)\right),$$

where $K$ is the dimension of latent space and ${w}_{kl}$ is the weight of KL-divergence loss given as a hyperparameter.

In addition, an adversarial loss term (${L}_{adv}$) is incorporated into the total loss function to correct batch effects inherent in the input data. Adversarial learning [55] is a technique to force model training towards increasing certain loss term values on purpose. Since the latent space should ideally contain cellular heterogeneity information correcting all batch effects, the ground-truth batch effect vectors should not be predictable from the embedded latent factors. This idea is applied to scMaui loss function in a way of increasing cross-entropy loss between ground-truth and predicted batch effect values. According to Eq. (1), the model tries to decrease ${L}_{total}$ by increasing ${L}_{adv}$. When total $J$ different batch effect factors are assigned, a cross-entropy loss ${L}_{adv}$ is calculated for each of those and summed up together at the end. This approach was proven to improve batch effect correction by VAE-based models in our previous work [24].

Furthermore, different types of batch effect factors are handled in parallel as separate input vectors of covariates and adversaries. Continuous variables are also supported for batch effect handling in the scMaui algorithm. These provide high flexibility in scMaui downstream analysis for single-cell multiomics data, while users have to preprocess the batch effect information fitting to the required format (e.g. one category of all integrated batch effect factors) when using the majority of other single-cell multiomics integration tools. If a one-hot encoded categorical batch effect factor ${b}^{j}$ containing $K$ labels is given, the adversarial learning uses a categorical cross-entropy loss function:

$${L}_{adv}({b}^{j}) ={-\Sigma }_{k=1...K}{{b}^{j}}_{k}log({{softmax(a}^{j}}_{k}(\mu_{joint}))),$$

where ${a}^{j}(\cdot )$ is the adversarial network assigned to the batch effect factor. We note that outputs of the adversarial network are softmax-normalised for the categorical cross-entropy loss. If the batch effect factor ${b}^{j}$ has continuous values, we use the squared error loss:

$${L}_{adv}({b}^{j}) ={\Sigma }_{k=1...K}({{b}^{j}}_{k}-{{a}^{j}}_{k}(\mu_{joint}){)}^{2},$$

where all notations are the same as the categorical cross-entropy loss function above.

#### Seamless handling of missing data

scMaui includes the unique benefit of handling missing data as well. Mosaic data where not all samples have complete modalities can be still modelled by scMaui using the “missingness” indicator. scMaui supports a “mask” layer where users can indicate which modalities or features are absent for each sample, so that loss can be calculated only with measured assays and the robust embedding space can be created despite of incomplete data, using the PoE-based calculation.

### Data preprocessing

Four different single-cell multiomics datasets in various biological scenarios were used to assess scMaui performance. From GEO accession number GSE194122 [25], we downloaded two single-cell bone marrow datasets: one was comprised of RNA-seq and ADTs data, whereas the other included RNA-seq and ATAC-seq. Mouse skin cell SHARE-seq data was downloaded from GEO accession number GSE140203 [26]. The skin cell data set contains RNA-seq and ATAC-seq profiles. Mouse embryo single-cell multiomics data, which consists of RNA-seq and BS-seq, was also collected with GEO accession number GSE121708 [45].

Since each assay has its own properties, different preprocessing pipelines were applied accordingly. For RNA-seq and ATAC-seq assays, sequencing read counts were normalised and *log1p*-transformed using scran [56] and scanpy [57]. Then, only for ATAC-seq assay, which is more sparse than RNA-seq, we excluded features covering less than 5% of the entire cells. ADTs were also normalised with the centred log-ratio transformation.

However, for the mouse skin SHARE-seq data, we mainly followed the preprocessing described by scMM paper [18]: choosing the top 5,000 genes and 25% peaks for RNA-seq and ATAC-seq, respectively. Features expressed in less than 10 cells were excluded before choosing the top features.

Only for ATAC-seq in the human bone marrow dataset (GSE194122) and BS-seq assays, we filtered out features included neither in promoters nor in enhancer regions. hg38 and mm10 genomes were used for finding promoter and enhancer regions, as a human and mouse reference genome each. Promoters were collected from UCSC dataset, while enhancers were downloaded from FANTOM5 (https://slidebase.binf.ku.dk/human_enhancers/, [58]). After the filtering, we selected only 5,000 most variable features from the assay.

The human bone marrow dataset (GSE194122) provides very detailed cell-type labels (45 labels for RNA-seq and ADTs data, 22 labels for RNA-seq and ATAC-seq data). Thus, we further coarsely annotated each cell by grouping given cell-type labels. In this study, the provided cell-type labels and the new group annotations are referred to as *subpopulation* and *population,* respectively. Supplementary Tables 1 and 2 show the annotation matches of population and subpopulation in individual datasets. For the benchmarking, we used 84,677 cells in total including 11 populations which comprise 45 subpopulations. These were collected from 8 different donors and processed in 4 sites, creating a multiple batch effect landscape representative of many real-world scenarios.

### Experimental setup

#### Assignment of batch effect factors

Depending on the single-cell multiomics data collection pipeline, different factors can introduce additional sources of variation within the dataset. For the human bone marrow dataset, two different factors were considered to cause batch effects: donor and site. On the other hand, in the mouse embryo development analysis, only different embryos were regarded as a batch effect factor. All the single-cell multiomics integration methods with the exception of scMaui and sciPENN took one merged vector of batches including both donor and site batch factors, because those accept only a single type of factor for batch effect correction.

#### scMaui

Although scMaui requires many different hyperparameters regarding VAE setup, we kept a consistent setup for most of those in all analyses. We gave 20 layers with 512 units to the encoder and 1 layer with 20 units to the decoder each. The adversary network was established with 2 layers consisting of 128 units. Only in the input layer, dropout was applied with the rate 0.1. We used 512 as batch size in all model training.

The number of latent factors and training epochs were chosen depending on the number of features within the dataset in each analysis. While the human bone marrow gene expression and protein expression analyses used only 28 latent factors due to a much lower number of protein expression features, the other 2 analyses were done with scMaui trained for 50 latent factors. All models were trained over 1500 epochs with the exception of the human bone marrow RNA-seq and ATAC-seq analysis whose training was 2000 epochs owing to the larger number of features and less sparsity in ATAC-seq data compared to the methylation data. Regarding the reconstrufction loss, we gave negative binomial loss to respective assays except for the BS-seq assay in the mouse embryo development analysis. Since methylation data generally presents distinct binary signals, methylated or unmethylated, in single-cell data, we utilised binary reconstruction error.

#### PCA

Principal component analysis (PCA) is a linear dimensionality reduction algorithm which extracts principal components that explain the most variance. We conducted PCA on each assay independently and extracted 20 components.

#### MOFA

MOFA [8] is an R package which can dissect multiomics datasets into integrated factors and provide a low dimensional reconstruction. In this study, MOFA2 version 1.6.0 was used and hyperparameters were chosen based on *get_default_model_options*, *get_default_data_options* and *get_defalut_training_options* functions provided by the package. MOFA also has the option to explain variations across different groups of samples, thus we did 2 separate analyses, with and without the group option, to compare the results in terms of batch effect correction.

#### Seurat

Seurat v3 [10] was mainly developed for integrating single-cell datasets generated via different experiments, but we used it in this study focusing more on its capacity to find associations between different assays. We applied *sctransform* only to the RNA-seq assay in all analyses. In order to handle the batch effect, the Seurat object was split into different batches. Then, we selected integration features with 3000 features, and found integration anchors between different assays. For finding integration anchors, we assigned 100 to the minimum number of neighbours as filtering threshold, ‘rann’ (it refers to as fast nearest neighbour search) to the neighbouring method. Ultimately, we ran PCA on the integrated data and extracted 20 components in each analysis.

In the mouse skin SHARE-seq benchmarking, more recently published versions of Seurat were applied. Seurat v4 introduced weighted-nearest neighbour (WNN) to find a joint distribution of multiple modalities of omics data to define cellular state [31], whereas a new pipeline to integrate scRNA-seq and scATAC-seq data was revealed in Seurat v5 tutorials [32]. In the Seurat v5 tutorial, Signac [59] is used to estimate the transcriptional activity based on ATAC-seq counts and the integration is done using canonical correlation analysis (CCA). For the benchmarking, we integrated the RNA-seq and ATAC-seq data from the mouse skin data set following these two pipelines in their tutorials (https://satijalab.org/seurat/archive/v4.3/weighted_nearest_neighbor_analysis and https://satijalab.org/seurat/articles/seurat5_atacseq_integration_vignette), separately. The results are annotated as *Seurat_wnn* and *Seurat_cca*, respectively.

#### totalVI and MultiVI

totalVI [9] is a VAE-based method for integrating specifically CITE-seq data, which is comprised of transcriptomes and epitopes, so it was only tested with the human bone marrow RNA-seq and ADTs data. We used totalVI included in python package scvi version 0.17.1. Batch label was given as the combination of donor and site. We set up batch size as 256, the ratio of training samples as 0.8 and the learning rate as $4\times {10}^{-3}$. The training was done over 300 epochs, and afterwards, we ran the posterior modelling with batch size 32. Otherwise, we followed up on all default hyperparameter setups described in the scvi-tools package tutorial (https://docs.scvi-tools.org/en/0.6.6/index.html).

MultiVI [30] is another VAE-based single-cell multiomcs integration method provided in the scvi-tools package. Since the targeted data types for the mouse SHARE-seq benchmarking (RNA-seq and ATAC-seq) are not supported by totalVI, we replaced totalVI with MultiVI. When it comes to hyperparameters and pipelines, we followed the tutorial provided by the authors (docs.scvi-tools.org/en/stable/tutorials/notebooks/multimodal/MultiVI_tutorial.html).

#### scMM

scMM [18] adopts the mixture-of-experts VAEs to integrate either RNA-seq and ATAC-seq or two modalities of CITE-seq. We downloaded the source code from the GitHub repository (https://github.com/kodaim1115/scMM) and used the *main.py* file for our benchmarking experiments. 30 and 40 latent factors were used for human bone marrow CITE-seq data and mouse skin SHARE-seq data each.

#### sciPENN

sciPENN [60] integrates CITE-seq data and imputes the missing values by primarily using feed-forward neural networks and recurrent neural networks. Since the method is specifically designed for the CITE-seq technology, we added sciPENN only in the human bone marrow CITE-seq data benchmarking. The baseline tutorial provided by the authors (https://github.com/jlakkis/sciPENN) was followed to run sciPENN for the benchmarking.

#### Mowgli

Mowgli [61] is a non-negative matrix factorisation (NMF) model combined with optimal transport for single-cell multiomics integration. Since Mowgli only performed reasonably with a lower number of features during the experiments, we selected the top 500 highly variable genes for RNA-seq in the human bone marrow CITE-seq data. For the mouse skin SHARE-seq data, we chose the top 800 features for each modality. The number of latent factors was 25 and 40 for the human bone marrow data and mouse skin SHARE-seq data integrations each.

### Performance measures

#### Louvain clustering

The Louvain community detection algorithm identifies clusters based on the relative density of connections between the inside and the outside of each cluster [62]. This method has been applied to a broad range of single-cell analyses [63]. We clustered latent factors with the Louvain clustering algorithm and assessed the performance of respective methods.

#### Adjusted mutual information

Mutual information (MI) measures the similarity between ground-truth clusters and predicted clusters. Given $n$ ground-truth clusters $U = \{{U}_{1}, {U}_{2}, ..., {U}_{n}\}$ and $m$ predicted clusters $V = \{{V}_{1}, {V}_{2}, ..., {V}_{m}\}$, MI between two clusters is given as:

$$MI\left(U, V\right)= {\sum }_{i=1}^{n}{\sum }_{j=1}^{m}\frac{\left|{U}_{i} {\cap }{V}_{j}\right|}{n + m} log\frac{\left(n+m\right)\left|{U}_{i} {\cap }{V}_{j}\right|}{\left|{U}_{i}\right|\left|{V}_{j}\right]}; \left|{X}_{t}\right|:= Number\, of \,elements \,in \,cluster {X}_{t}.$$

However, MI value tends to be higher when the number of clusters is larger. Therefore, we used adjusted mutual information (AMI) in our evaluation, which takes account of chance as follows:

$$AMI(U,V) =\frac{MI(U, V) - E(MI(U, V))}{max(MI(U, V)) - E(MI(U, V){)}}; E(x) = Expectation\, of\, x.$$

#### Adjusted rand index

Rand index (RI) is a statistic measuring the similarity between two different clusters. In our analysis, it was used for comparing predicted clusters and ground-truth clusters. RI is calculated as follows:

$$RI = \frac{TP + TN}{TP + FP + FN + TN}$$

where $TP , TN, FP, FN$ refer to true positive, true negative, false positive, and false negative, respectively. Adjusted rand index (ARI) is the corrected version of RI for chance and makes the measurement independent of the number of clusters. ARI equation is defined as:

$$ARI =\frac{RI - E(RI)}{max(RI) - E(RI{)}}; E(x) = Expectation\ of\ x.$$

#### Clustering purity

We also calculated the purity of the most dominant class in each cluster. When $C$ different ground-truth classes are grouped into one cluster, the clustering purity is calculated as follows:

$$Clustering\, Purity = \frac{max({n}_{i})}{{\prod }_{i=1}^{C}{n}_{i}}; i = 1, 2, ..., C.$$

${n}_{i}$ means the number of elements from the class $i$.

#### Silhouette score

For silhouette score values, we used the silhouette coefficient which measures the similarity of each element to the cluster where it belongs, compared to the closest neighbouring cluster. In single-cell analysis, it often represents the separation of clusters meaning that higher value indicates better separation. For each cell ${c}_{i}$, the silhouette score is calculated as below:

$$Silhouette Score = \frac{b({c}_{i}) - a({c}_{i})}{max(b({c}_{i}) ,a({c}_{i}))}$$

where $b({c}_{i})$ refers to the mean distance between the given cell ${c}_{i}$ and the nearest neighbouring cluster, and, $a({c}_{i})$ is the mean distance between the given cell ${c}_{i}$ and all other cells within the same cluster.

### Supplementary Information

Supplementary Material 1.

## Data Availability

Both human bone marrow and mouse embryo single-cell multiomics datasets are available on GEO data repository with GEO accession number GSE194122 (https://ncbi.nlm.nih.gov/geo/query/acc.cgi?acc=GSE194122), GSE140203 (https://ncbi.nlm.nih.gov/geo/query/acc.cgi?acc=GSE140203), and GSE121708 (https://ncbi.nlm.nih.gov/geo/query/acc.cgi?acc=GSE121708) each.

## Code availability

scMaui code is available on our GitHub repository (https://github.com/BIMSBbioinfo/scmaui). The GitHub page also provides command-line usage. The tutorials for different types of multi-omics data sets are provided on another GitHub repository (https://github.com/BIMSBbioinfo/scmaui-experiments).
