# Supplementary material for: scMaui: a widely applicable deep learning framework for single-cell multiomics integration in the presence of batch effects and missing data
Source: BMC Bioinformatics. 2024 Aug 6;25:257. doi: 10.1186/s12859-024-05880-w (PMC11304929; doi:10.1186/s12859-024-05880-w)
Supplement: Supplementary file 1 — Supplementary Material 1. [file 12859_2024_5880_MOESM1_ESM.pdf]

Supplementary Information

Methods

Reconstruction loss functions in scMaui

Here, we assume that the input assay (vector) Y is comprised of N features, $Y = [y_1, y_2, \dots, y_N]$, and accordingly, the scMaui decoder outputs the reconstructed assay (vector), $\hat{Y} = [\hat{y}_1, \hat{y}_2, \dots, \hat{y}_N]$ with N features. In the loss functions using the binomial distribution, we specifically applied the sigmoid function $S(x)$ to the output logit of the decoder as an activation function. The sigmoid function is defined as:

$$S(x) = 1/(1 + e^{-x}).$$

Poisson loss

Poisson distribution, which originally models the probability that an event occurs a given number of times, is broadly used for omics data based on read counts. scMaui uses the negative log-likelihood of Poisson distribution to calculate the Poisson reconstruction loss as follows:

$$L_{poisson} = -\frac{1}{N}\sum_{i=1}^{N}(y_i log(\hat{y}_i) - \hat{y}_i).$$

Negative binomial and negative multinomial distribution losses

Due to the greater value of variance than the mean value, the negative binomial distribution is considered a better model for overdispersion than the Poisson distribution. For this reason, it is commonly used for explaining overdispersion in gene expression count data[1,2]. Thus, scMaui supports the negative log-likelihood of negative binomial distribution with parameters r and p, as a reconstruction loss:

$$L_{negbinom} = -\frac{1}{N}\sum_{i=1}^{N}(log\Gamma(y_i + r) - log\Gamma(r) - log\Gamma(y_i + 1) + y_i log(S(\hat{y}_i)) + rlog(S(-\hat{y}_i))).$$

Since p, which refers to the probability of success in the binomial distribution, is assumed to be between 0 and 1 in a negative binomial distribution, we converted the logit value outputted from the decoder with the sigmoid function

On the other hand, in our previous work, negative multinomial distribution reconstruction loss outperformed negative binomial distribution loss with binarised single-cell ATAC-seq assay [3]. Therefore, we included it in the scMaui package following the implementation in the previous work:

$$L_{negmul} = -log\Gamma(\sum_{i=1}^{N} y_i + r) + log\Gamma(r) - rlog(P_0) + \sum_{i=1}^{N} y_i log(P_i).$$

P_0 and P_i are softmax-modified functions to make the non-negative parameters of multinomial distribution from the output of decoder $\hat{y}_i$. P_i reflects each feature of the input assay y_i as follows:

$$P_i = \frac{exp(\hat{y}_i)}{1+\sum_{i=1}^{N} exp(\hat{y}_i)}.$$

P_0 is used for explaining the dispersion in the data as below:

$$P_0 = \frac{1}{1+\sum_{i=1}^{N} exp(\hat{y}_i)}.$$

Binary loss

Some single-cell omic assays, such as BS-seq or binarised ATAC-seq, resemble a bimodal distribution, so scMaui provides the binary loss function to reconstruct this kind of assays. It is based on binomial distribution which models the number of successes in a sample size. The loss function again uses the negative log-likelihood of binomial distribution with a parameter n, the number of total trials, defined as:

$$L_{binary} = -\frac{1}{N}\sum_{i=1}^{N}(y_i log(S(\hat{y}_i))_- + (n_i - y_i) log(S(-\hat{y}_i))).$$

Due to the same reason as explained in negative binomial loss, we converted the output logit with the sigmoid function.

MSE and MAE

Mean squared error (MSE) and mean absolute error (MAE) calculate the error between the ground truth and the reconstruction directly without any distribution:

$$L_{MSE} = \frac{1}{N}\sum_{i=1}^{N}(y_i - \hat{y}_i)^2,$$

$$L_{MAE} = \frac{1}{N}\sum_{i=1}^{N}|y_i - \hat{y}_i|.$$

References

1. Durán Pacheco, Gonzalo, et al. "Performance of analytical methods for overdispersed counts in cluster randomized trials: Sample size, degree of clustering and imbalance." *Statistics in medicine* 28.24 (2009): 2989-3011.
2. Li, Qian, et al. "Subject level clustering using a negative binomial model for small transcriptomic studies." *BMC bioinformatics* 19.1 (2018): 1-10.
3. Kopp, Wolfgang, Altuna Akalin, and Uwe Ohler. "Simultaneous dimensionality reduction and integration for single-cell ATAC-seq data using deep learning." *Nature Machine Intelligence* 4.2 (2022): 162-168.

Subpopulation	Population	Subpopulation	Population
Naive CD20+ B IGKC+	B	ILC1	ILC
Naive CD20+ B IGKC-		ILC	
B1 B IGKC+		Plasma cell IGKC+	Plasma
B1 B IGKC-		Plasma cell IGKC-	
Transitional B		CD4+ T naive	T
CD14+ Mono	Mono	CD4+ T activated	
CD16+ Mono		CD4+ T activated integrinB7+	
HSC	HSC	CD4+ T CD314+ CD45RA+	
Reticulocyte	Reticulocyte	CD8+ T naive	
Normoblast	Blast	CD8+ T CD49f+	
Erythroblast		CD8+ T TIGIT+ CD45RO+	
Plasmablast IGKC+		CD8+ T CD57+ CD45RA+	
Plasmablast IGKC-		CD8+ T CD69+ CD45RO+	
Proerythroblast		CD8+ T TIGIT+ CD45RA+	
NK CD158e1	NK	CD8+ T CD69+ CD45RA+	
NK		CD8+ T naive CD127+ CD26- CD101-	
pDC	Dendritic	CD8+ T CD57+ CD45RO+	
cDC2		MAIT	
cDC1		T reg	
Lymph prog	Prog	gdT TCRVD2+	
G/M prog		gdT CD158b+	
MK/E prog		dnT	
T prog cycling			

Supplementary Table 1. *Cell-type labels (subpopulation) and newly annotated population labels in GSE194122 single-cell gene and protein expression multiomics dataset*

<table>
<tr><th>Subpopulation</th><th>Population</th><th>Subpopulation</th><th>Population</th></tr>
<tr><td>Naive CD20+ B</td><td rowspan="3">B</td><td>CD8+ T</td><td rowspan="4">T</td></tr>
<tr><td>B1 B</td><td>CD8+ T Naive</td></tr>
<tr><td>Transitional B</td><td>CD4+ T naive</td></tr>
<tr><td>CD14+ Mono</td><td rowspan="2">Mono</td><td>CD4+ T activated</td></tr>
<tr><td>CD16+ Mono</td><td>Lymph prog</td><td rowspan="4">Prog</td></tr>
<tr><td>Erythroblast</td><td rowspan="3">Blast</td><td>G/M prog</td></tr>
<tr><td>Normoblast</td><td>MK/E prog</td></tr>
<tr><td>Proerythroblast</td><td>ID2-hi myeloid prog</td></tr>
<tr><td>NK</td><td>NK</td><td>pDC</td><td rowspan="2">Dendritic</td></tr>
<tr><td>ILC</td><td>ILC</td><td>cDC2</td></tr>
<tr><td>HSC</td><td>HSC</td><td>Plasma</td><td>Plasma</td></tr>
</table>

Supplementary Table 2. *Cell-type labels (subpopulation) and newly annotated population labels in GSE194122 single-cell gene expression and ATAC-seq multiomics dataset*

	Baseline model	Available multiomics modality			
		Gene expression	Chromatin accessibility	Protein expression	Methylation
MOFA	Variational inference	✓	✓	✓	✓
Seurat	CCA or WNN[1]	✓	✓	✓	✓
totalVI	VAE[2]	✓		✓	
MultiVI	VAE	✓	✓	✓	
scMM	VAE	✓	✓	✓	
sciPENN	FNN and RNN[3]	✓		✓	
Mowgli	NMF[4]	✓	✓	✓	
**scMaui**	**VAE**	✓	✓	✓	✓

Supplementary Table 3. *Available multiomics modalities for each benchmarked single-cell multiomics integration method*

[1] Canonical Correlation Analysis or Weighted-nearest Neighbour
[2] Variational Autoencoder
[3] Feed-forward neural network and Recurrent neural network
[4] Non-negative matrix factorisation

Subpopulation	Best performing method	Subpopulation	Best performing method
B1 B	**scMaui**	ILC	scMM
CD14+ Mono	scMM	Lymph prog	GEX+PCA
CD16+ Mono	GEX+PCA	MK/E prog	GEX+PCA
CD4+ T activated	**scMaui**	NK	MOFA
CD4+ T naive	GEX+PCA	Naive CD20+ B	**scMaui**
CD8+ T	Seurat	Normoblast	MOFA
CD8+ T naive	GEX+PCA	Plasma cell	scMM
Erythroblast	MOFA	Proerythroblast	Seurat
G/M prog	MOFA	Transitional B	MOFA
HSC	Seurat	cDC2	**scMaui**
ID2-hi myeloid prog	**scMaui**	pDC	MOFA

Supplementary Table 4. *Best performing method for each subpopulation classification in GSE194122 single-cell gene expression and ATAC-seq multiomics dataset*

Batch effect handling	Population classification mean AUC	Population silhouette score	Batch silhouette score
Without batch effect handling	0.985	0.131	0.022
Only adversaries	0.992	**0.258**	-0.036
Only covariates	0.989	0.132	-0.030
Both	**0.993**	0.253	**-0.050**

Supplementary Table 5. *scMaui population classification and batch handling performances for different batch handling strategies.*

Model	Silhouette score	AMI	ARI	Best resolution	Clustering purity	# Clusters
GEX+PCA	0.061	**0.626**	**0.458**	0.8	0.738	15
ATAC+PCA	0.012	0.516	0.356	0.7	0.588	15
scMaui	0.056	0.605	0.389	1.5	0.738	24
MOFA	0.006	0.532	0.375	0.4	0.619	12
Seurat_cca	0.040	0.604	0.392	1.7	0.730	24
Seurat_wnn	**0.195**	0.666	0.467	0.1	**0.788**	20
MultiVI	0.109	0.571	0.333	0.9	0.678	27
scMM	0.074	0.562	0.347	0.8	0.681	24
Mowgli	0.009	0.417	0.222	1.4	0.563	26

Supplementary Table 6. *Performance comparison with respect to clustering and dimensionality reduction for mouse skin SHARE-seq data set. Louvain clustering algorithm was applied to the low-dimensional latent factors/features extracted by each method. The best value and the second best value of each score are highlighted in bold and underlined, respectively.*

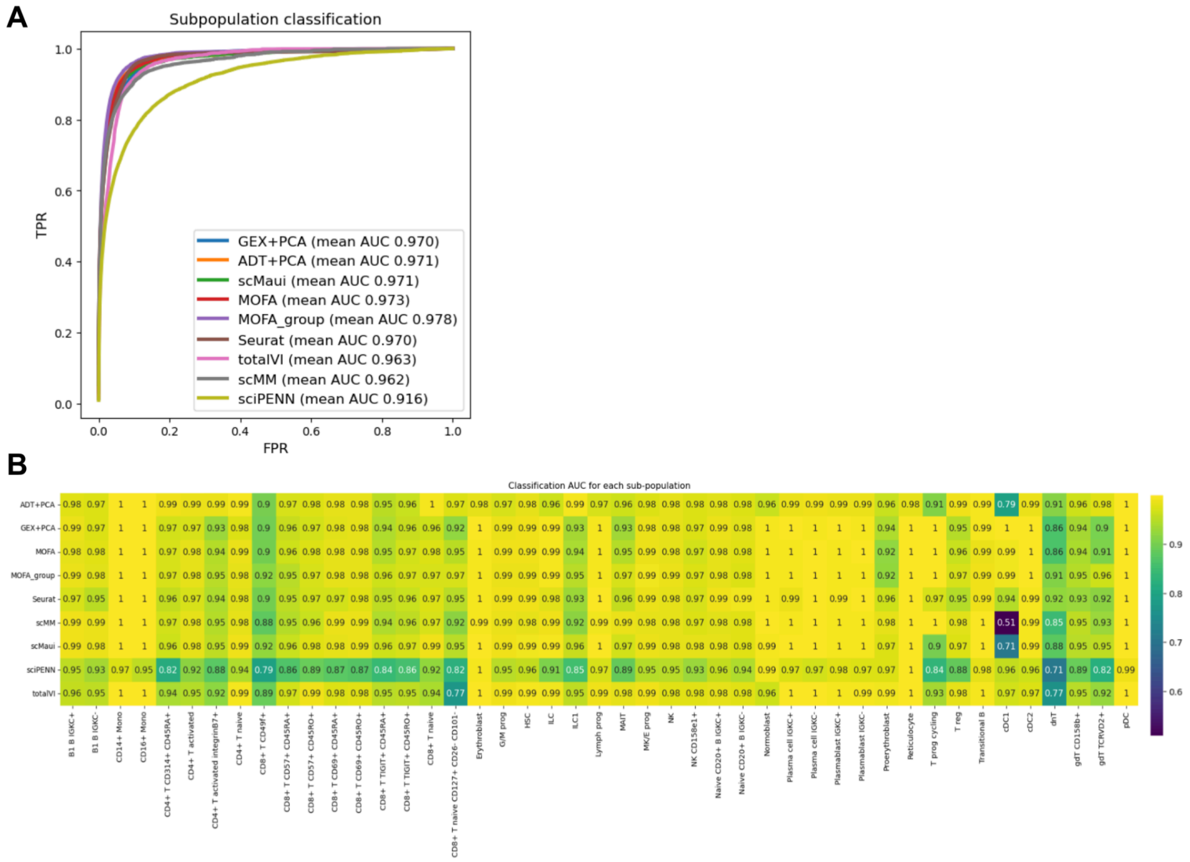

Supplementary Figure 1. *Cell subpopulation classification results.* ***A.*** *Cell subpopulation ROC curves and mean AUC.* ***B.*** *Classification AUC value for each subpopulation and each method.*

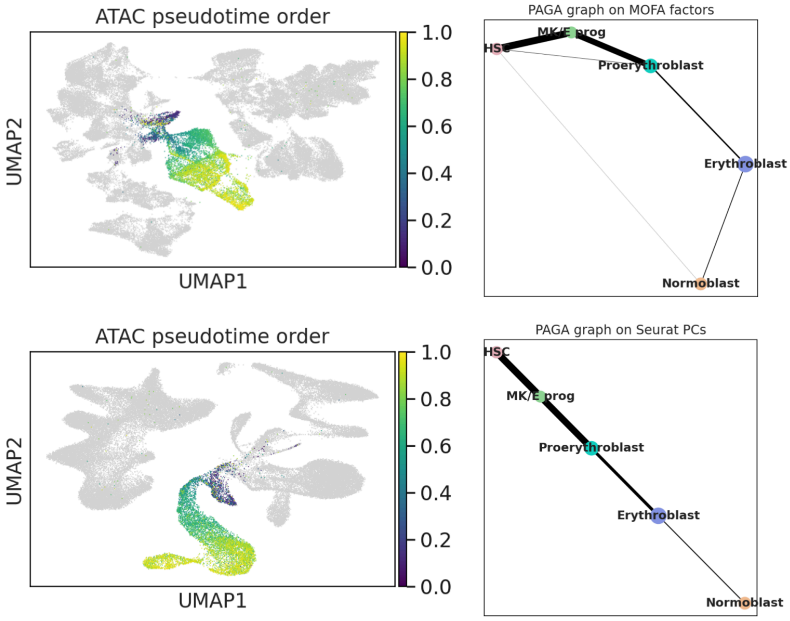

Supplementary Figure 2. *ATAC pseudotime order representation on UMAP plots and inferred PAGA graphs of MOFA (top) and Seurat (bottom).*

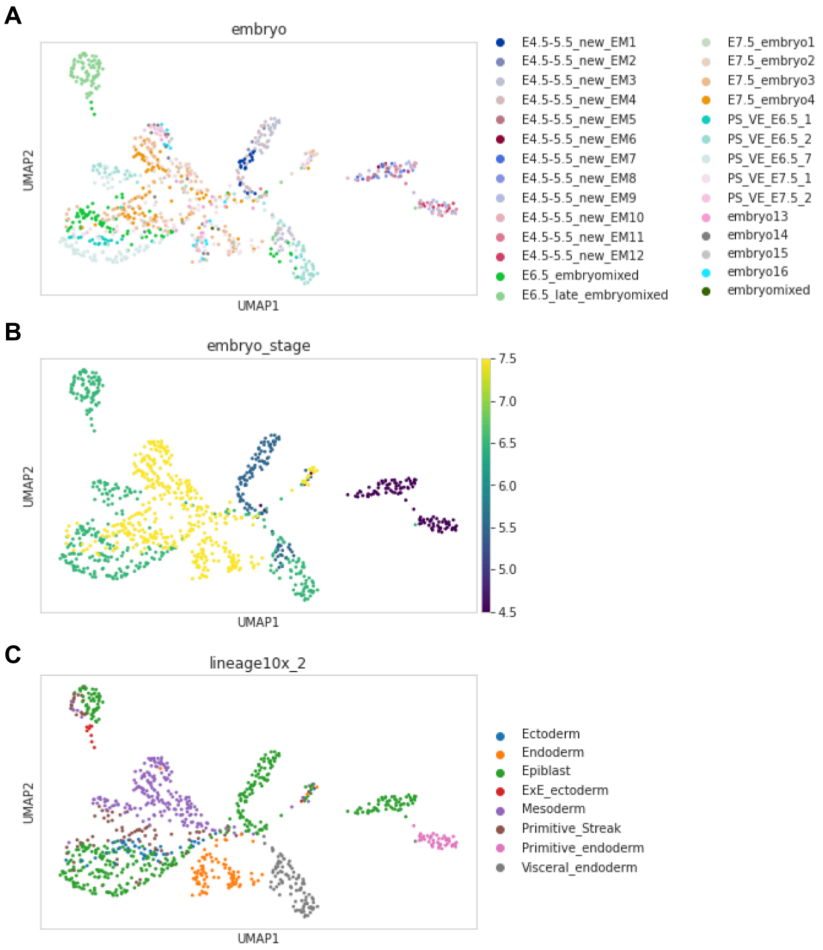

Supplementary Figure 3. *UMAP plot of 20 principal components extracted from mouse embryo gene expression assay.* ***A.*** *UMAP coloured by embryo samples* ***B.*** *UMAP coloured by embryo stages* ***C.*** *UMAP coloured by cell-types*

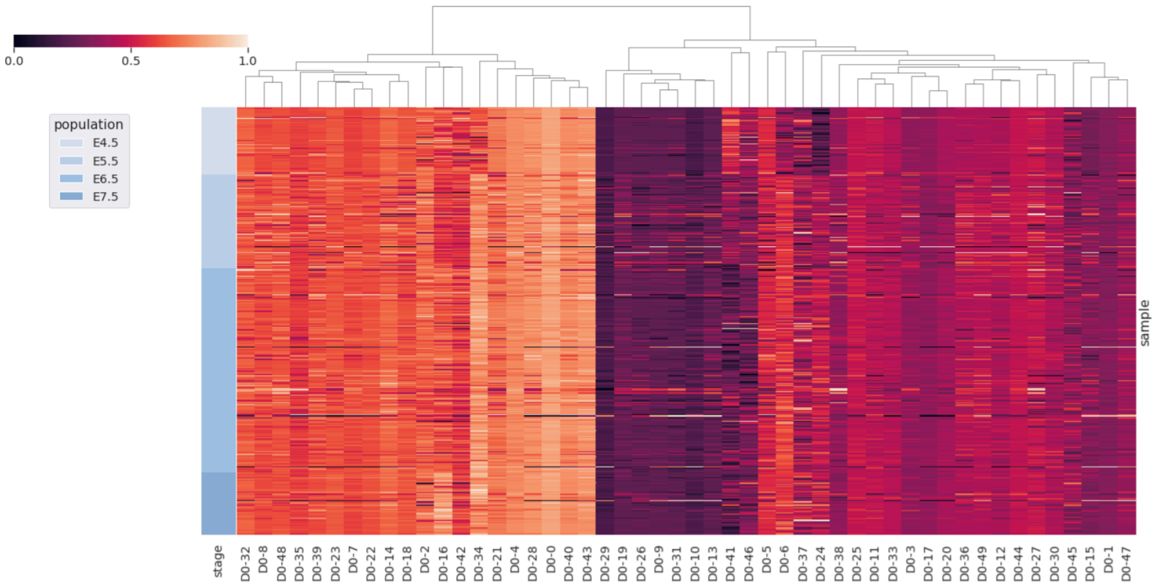

Supplementary Figure 4. *scMaui latent values normalised between 0 and 1 and ordered by the embryo development stage.*

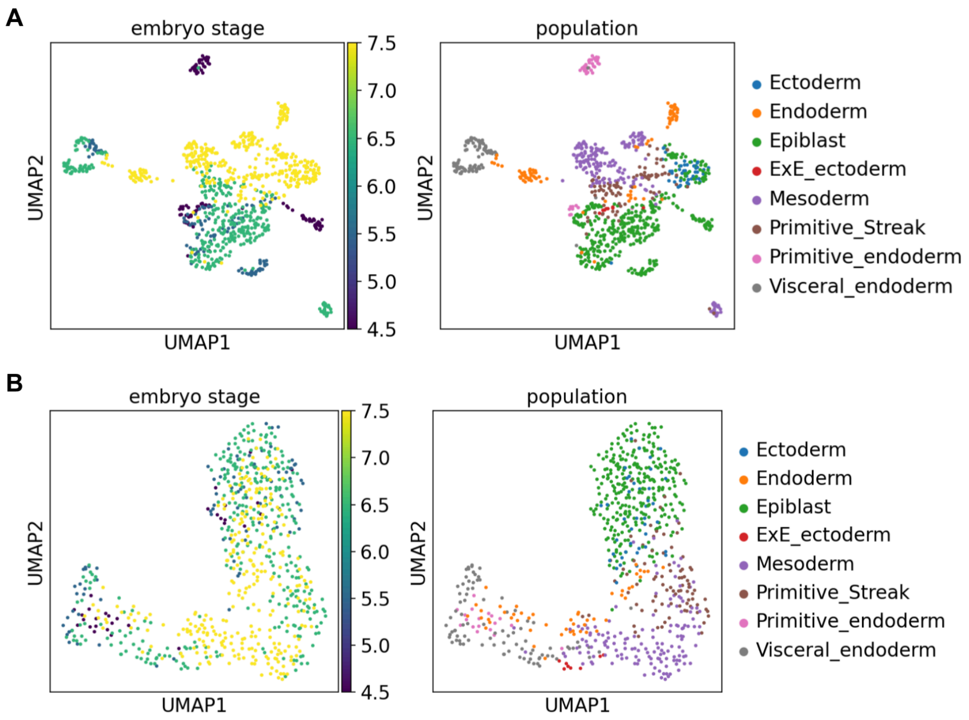

Supplementary Figure 5. *UMAP plot of MOFA factors (A) and Seurat PCs (B) coloured by embryo stage and population*

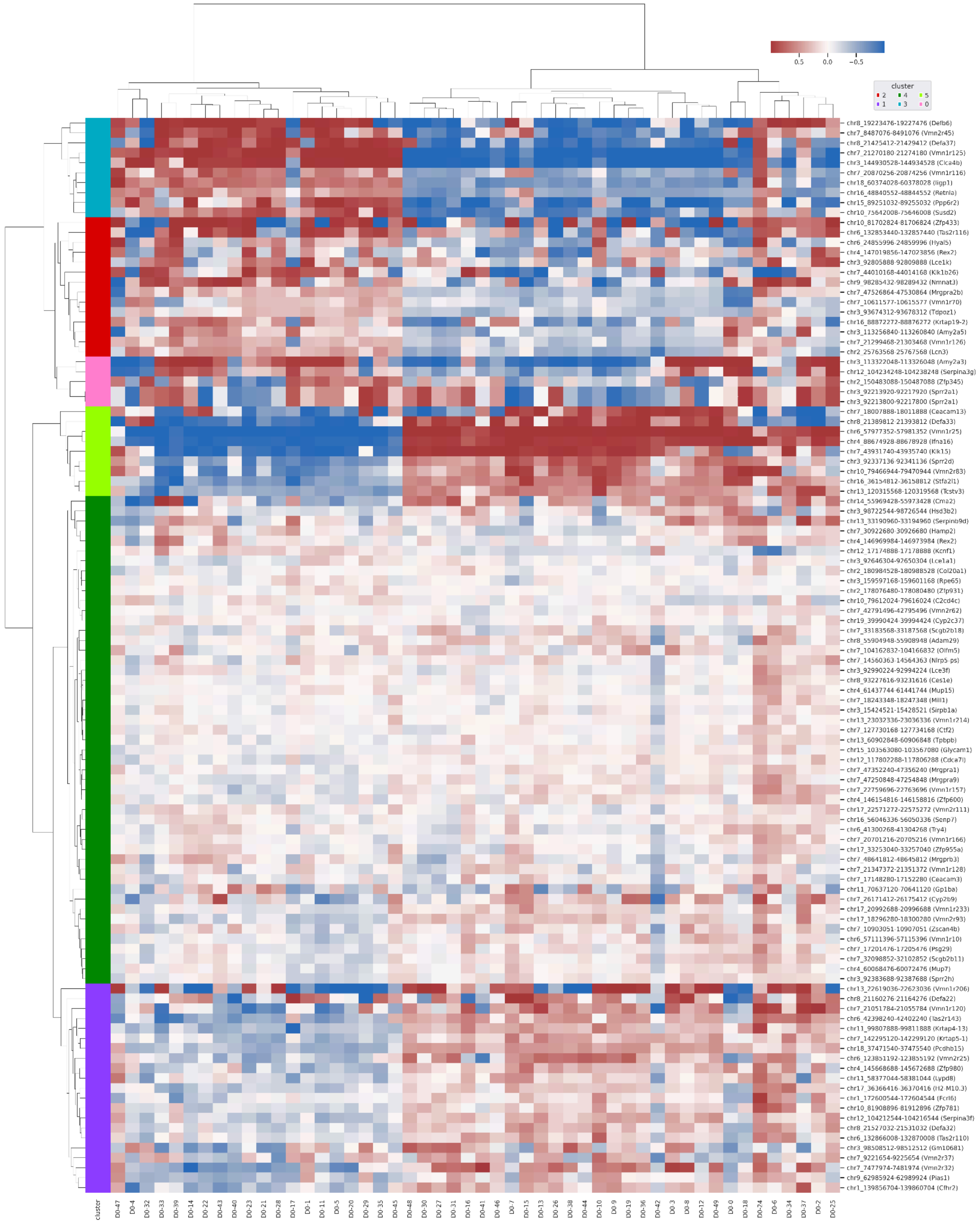

Supplementary Figure 6. *Correlation between methylation level in promoter/enhancer regions and scMaui latent factors. Based on the correlation, we grouped regions into six clusters using the agglomerative hierarchical clustering method.*

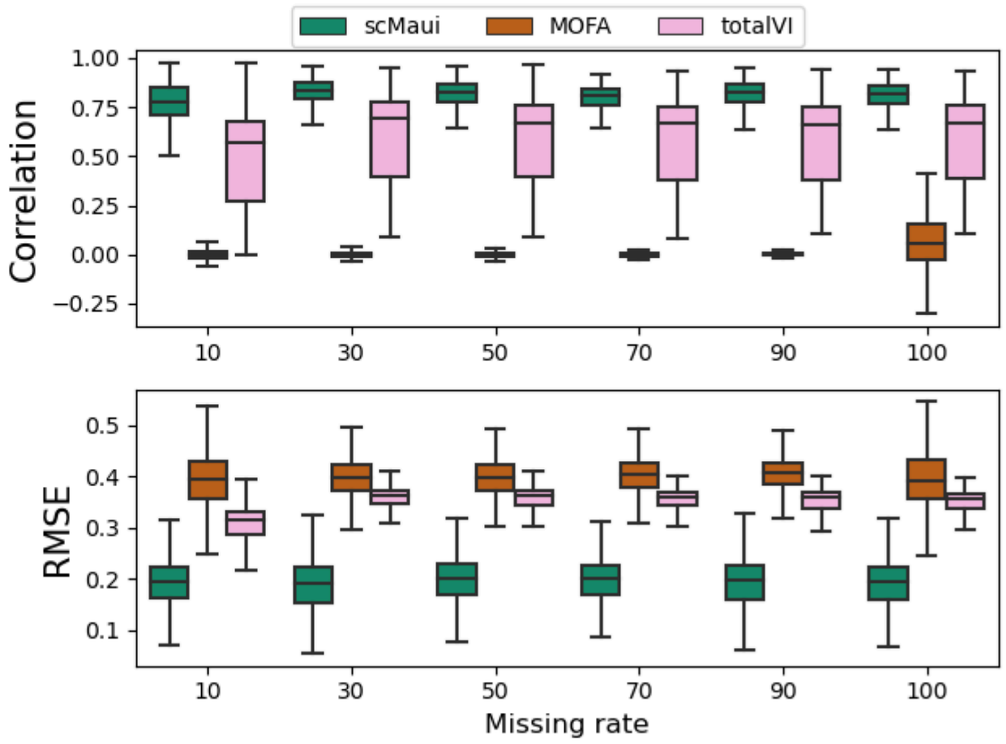

Supplementary Figure 7. *Gene expression modality imputation results. Correlation (top) and RMSE (bottom) values were calculated between the ground truth and the estimated expression levels.*

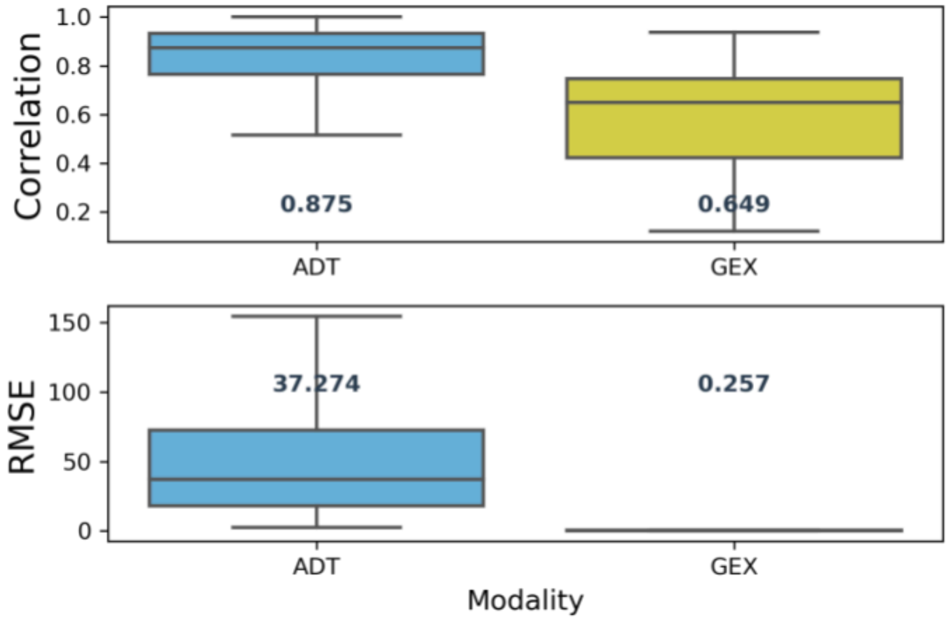

Supplementary Figure 8. *scMaui imputation performance when both gene and protein expression modalities were masked. Correlation (top) and RMSE (bottom) values were calculated between the ground truth and the estimated expression levels. The grey number at each box indicates the median value.*

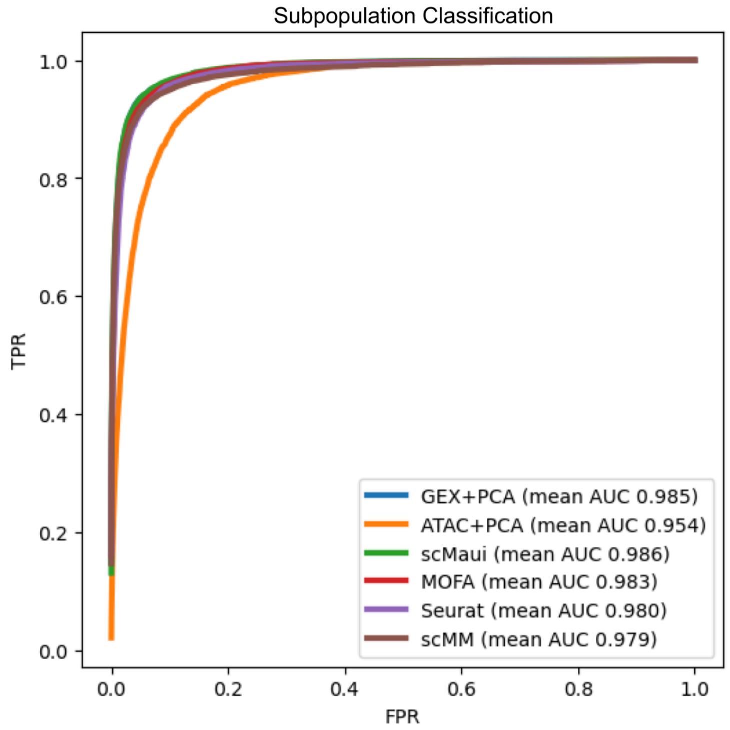

Supplementary Figure 9. *Cell subpopulation classification ROC curves and mean AUC values for single-cell gene expression and ATAC-seq integration.*

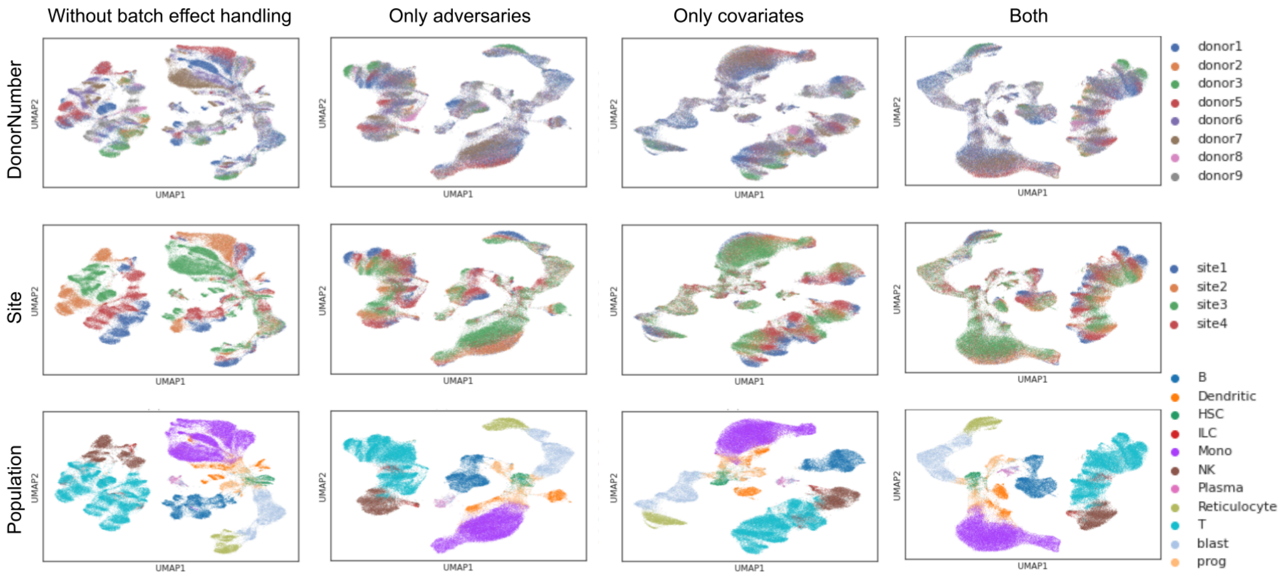

Supplementary Figure 10. *UMAP representation of scMaui latent factors calculated with different batch handling strategies. For each strategy, the UMAP plots are coloured by two batch effect factors (donors and sites) and cell population labels.*

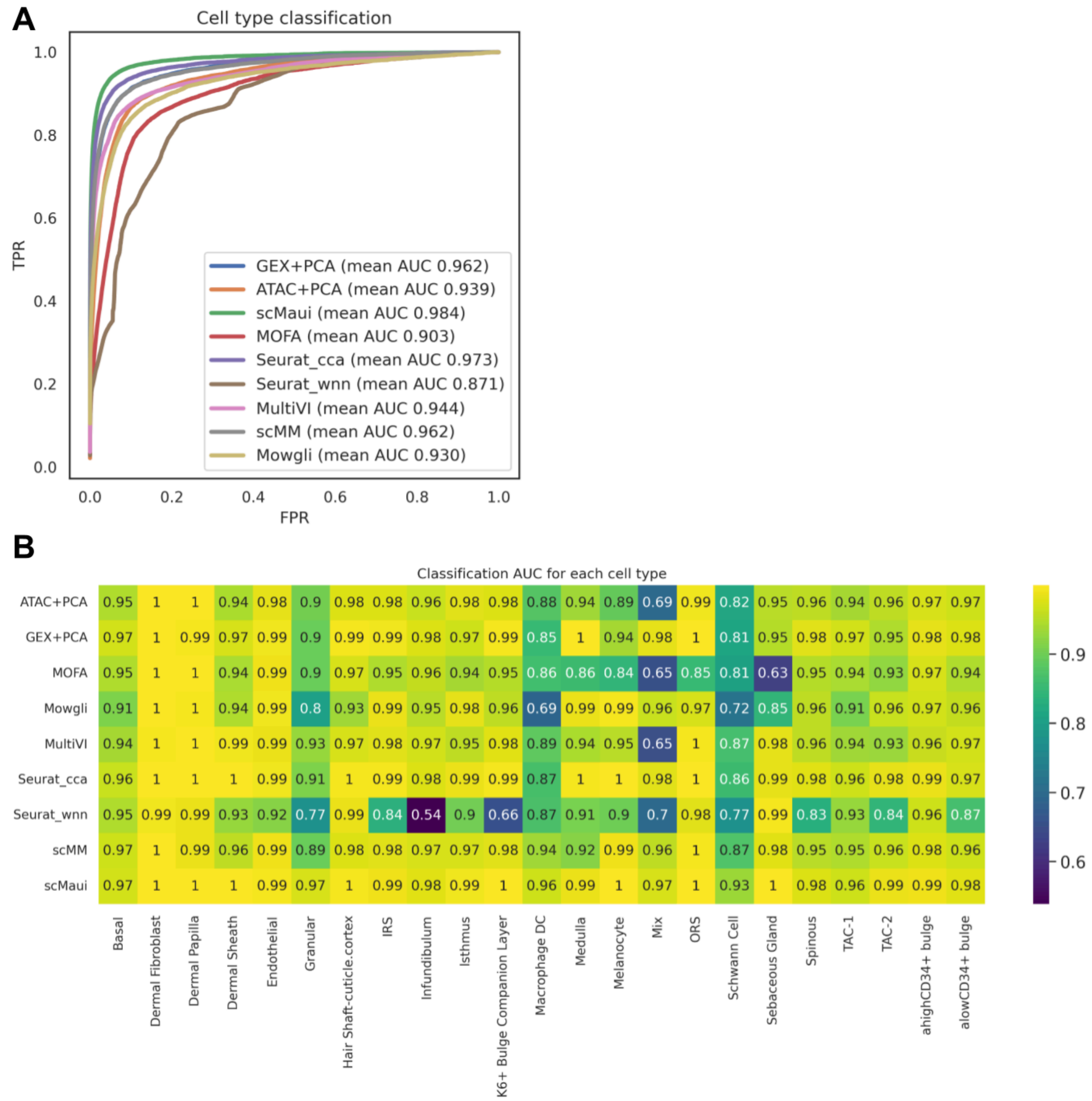

Supplementary Figure 11. *Cell-type classification results for mouse skin SHARE-seq data set.* ***A.*** *Cell-type ROC curves and mean AUC.* ***B.*** *Classification AUC value for each cell type and each method.*

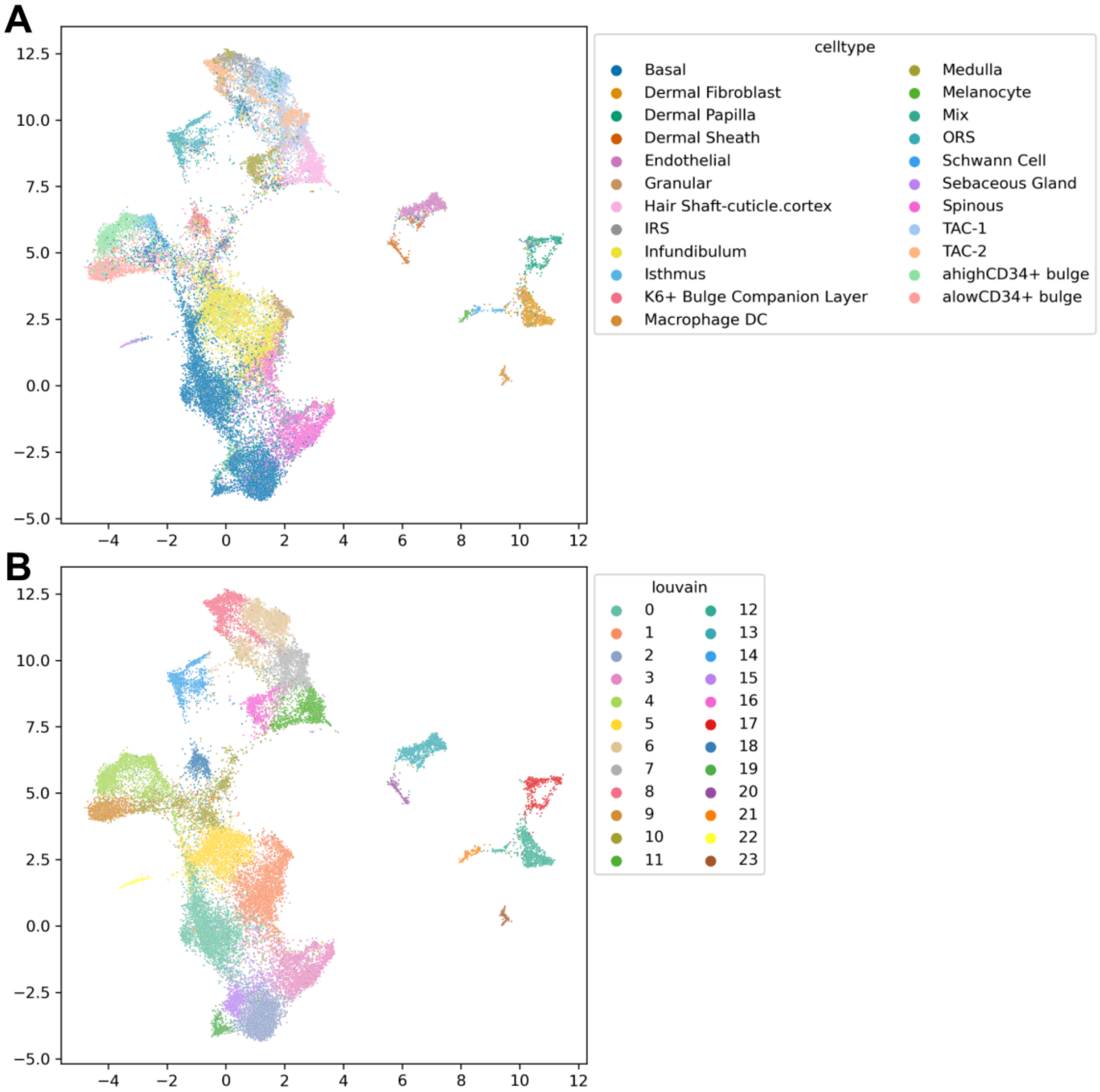

Supplementary Figure 12. *UMAP representation of scMaui latent factors for mouse skin SHARE-seq data set. Cells are coloured by **A.** ground-truth cell-type labels and **B.** Louvain clustering results.*

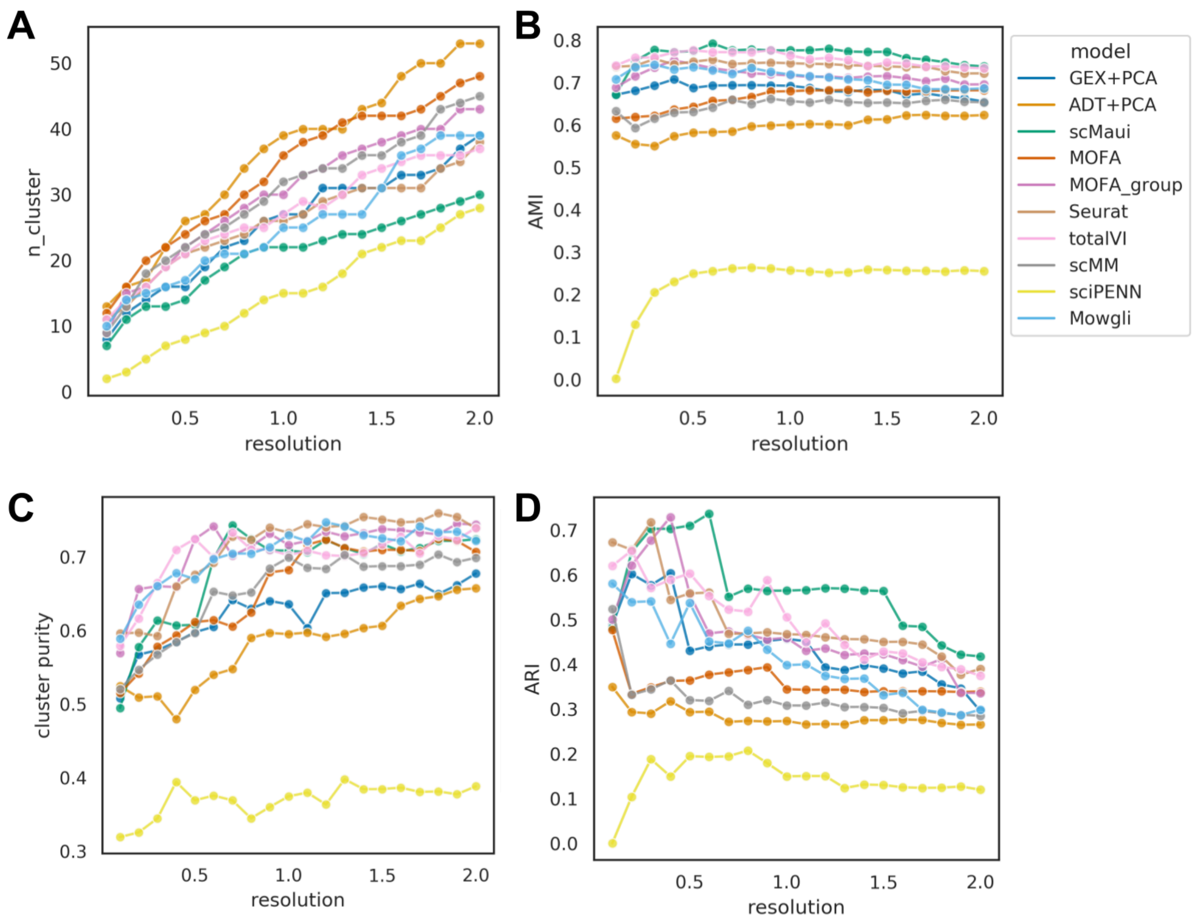

Supplementary Figure 13. *Cell-type clustering performance comparison over different resolution values for the Louvain clustering algorithm.* ***A.*** *Number of detected clusters.* ***B.*** *Adjusted mutual information.* ***C.*** *Clustering purity.* ***D.*** *Adjusted random index.*
